# Novel codon-optimized mini-intronic plasmid for efficient, inexpensive, and xeno-free induction of pluripotency

**DOI:** 10.1038/srep08081

**Published:** 2015-01-28

**Authors:** Sebastian Diecke, Jiamiao Lu, Jaecheol Lee, Vittavat Termglinchan, Nigel G. Kooreman, Paul W. Burridge, Antje D. Ebert, Jared M. Churko, Arun Sharma, Mark A. Kay, Joseph C. Wu

**Affiliations:** 1 Institute for Stem Cell Biology and Regenerative Medicine; Stanford University School of Medicine, Stanford, California 94305, USA; 2 Stanford Cardiovascular Institute; Stanford University School of Medicine, Stanford, California 94305, USA; 3 Department of Medicine, Division of Cardiology; Stanford University School of Medicine, Stanford, California 94305, USA; 4 Departments of Pediatrics and Genetics; Stanford University School of Medicine, Stanford, California 94305, USA; 5 Max Delbrück Center, Robert-Rössle Strasse 10, 13125 Berlin, Germany; 6 Berlin Institute of Health, Kapelle-Ufer 2, 10117 Berlin, Germany

## Abstract

The development of human induced pluripotent stem cell (iPSC) technology has revolutionized the regenerative medicine field. This technology provides a powerful tool for disease modeling and drug screening approaches. To circumvent the risk of random integration into the host genome caused by retroviruses, non-integrating reprogramming methods have been developed. However, these techniques are relatively inefficient or expensive. The mini-intronic plasmid (MIP) is an alternative, robust transgene expression vector for reprogramming. Here we developed a single plasmid reprogramming system which carries codon-optimized (Co) sequences of the canonical reprogramming factors (Oct4, Klf4, Sox2, and c-Myc) and short hairpin RNA against p53 ("4-in-1 CoMiP"). We have derived human and mouse iPSC lines from fibroblasts by performing a single transfection. Either independently or together with an additional vector encoding for LIN28, NANOG, and GFP, we were also able to reprogram blood-derived peripheral blood mononuclear cells (PBMCs) into iPSCs. Taken together, the CoMiP system offers a new highly efficient, integration-free, easy to use, and inexpensive methodology for reprogramming. Furthermore, the CoMIP construct is color-labeled, free of any antibiotic selection cassettes, and independent of the requirement for expression of the Epstein-Barr Virus nuclear antigen (EBNA), making it particularly beneficial for future applications in regenerative medicine.

The groundbreaking studies of Shinya Yamanaka and James Thomson showed that certain combinations of different pluripotency and oncogenic transcription factors (*OCT4, SOX2, NANOG*, *MYC*, and *LIN28*) were able to initiate the molecular circuitry of pluripotency and convert somatic cells into induced pluripotent stem cells (iPSCs)^1^,^2^. Based on morphology, growth characteristics, and gene expression profiles, these iPSCs are nearly indistinguishable from embryonic stem cells (ESCs)^3^,^4^. Like ESCs, iPSCs have the potential to differentiate into all three germ layers of the human body, opening new opportunities for drug discovery approaches and stem cell-based therapies. The first iPSCs were derived using viruses that integrate into the host genome, potentially altering the gene expression of the target cell^1^,^5^. To circumvent the subsequent risk of oncogene mutation and potential tumor formation, various non-integrating reprogramming techniques using Sendai virus, mRNAs, and protein- or DNA-based methods have been developed^6^,^7^,^8^,^9^,^10^. Because mRNA and protein-dependent methods are labor intensive and have a low reprogramming efficiency, most laboratories currently use Sendai virus or different episomal plasmids for the induction of pluripotency. Although Sendai virus-mediated iPSC conversion is the most efficient non-integrating reprogramming technique, there are considerable drawbacks such as high costs and viral biosafety concerns that must be addressed. To that end, we focused on developing an alternative, inexpensive, and user-friendly episomal reprogramming method.

We have recently shown that using a special episomal vector devoid of any bacterial plasmid backbone is an alternative method to generate transgene-free iPSCs from human adult cells^6^,^11^,^12^. These so-called minicircles are smaller in size than standard plasmids, which also enhance their transfection and expression rate. To produce high-quality minicircle DNA, we used a new simple, rapid, and inexpensive minicircle production system^13^. However, because the minicircle production and purification methodology is relatively complex and time consuming compared to viral methods, we aimed to develop a novel and user-friendly reprogramming plasmid to overcome these drawbacks.

Recently, a mini-intronic plasmid (MIP) vector has been reported as an alternative, robust, non-silencing transgene expression DNA vector *in vitro* and *in vivo*^14^. The defining feature of MIP vectors is the presence of pUC replication origin and the RNA-OUT antibiotic-free selectable marker in a designated 5′ noncoding exon. The MIP vector overcomes the transgene silencing observed with plasmids and provides at least 2–10 times higher levels of transgene expression compared to a minicircle vector containing the same expression cassette *in vitro* and *in vivo*^14^. To convert a differentiated cell into a pluripotent state, it is necessary to express high levels of the reprogramming factors with the right stoichiometry within a transfected cell^15^. The usage rates of codons vary among different organisms and genes, which in turn can influence the levels of protein expression^16^. Codon optimization increases protein expression by substitution of rare codons with more frequent codons in the amino acid sequence of the protein of interest.

Therefore, our objective here was to investigate the possibility of using a MIP vector backbone together with the codon optimized (Co) reprogramming factors to induce pluripotency efficiently. We introduced a codon-optimized polycistronic expression cassette, in which the four reprogramming factors are controlled by one promoter separated by different self-cleaving 2a peptide sequences and are integrated into a MIP vector. By combining two independent techniques, we were able to generate a novel, single plasmid reprogramming system called CoMiP, which is highly efficient, integration-free, and cost-effective.

## Results

### Higher transfection and expression efficiencies by using the 4-in-1 CoMiP episomal plasmid

**Figure 1:**
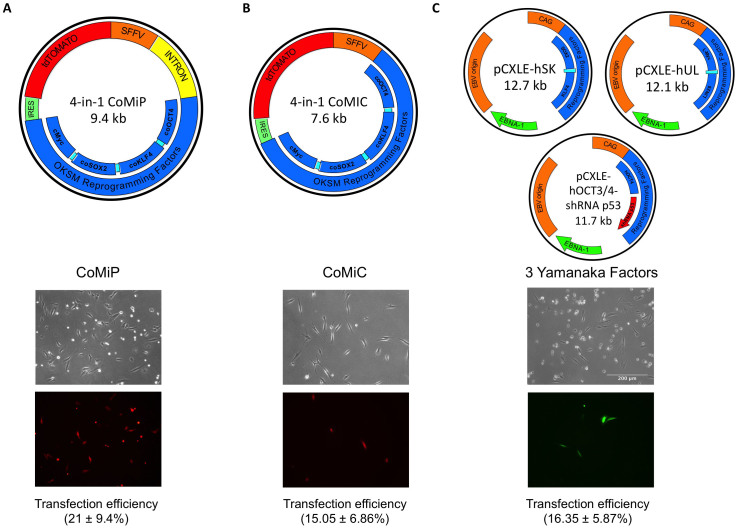
Side-by-side comparison of 3 different reprogramming techniques. The reprogramming vectors include (A) 4-in-1 CoMiP, (B) 4-in-1 Minicircle, and (C) 3 Yamanaka episomal plasmids. The highest transfection, expression efficiencies, and survival rate were observed with the 4-in-1 CoMiP reprogramming plasmid (A). Lower but moderate transfection efficiency and cell survival rates were observed by using either minicircle (B) or episomal (C) reprogramming plasmids.

In our previous study, we showed that the transfection and expression efficiencies of episomal plasmids are dependent on their overall size and the length of the DNA sequence required for their bacterial amplification^17^. Therefore, we first compared the transfection efficiency of our novel 4-in-1 CoMiP plasmids with already established reprogramming techniques, namely minicircle and EBNA/OriP-based episomal plasmids carrying the canonical Yamanaka factors^6^,^9^. Because the EBNA/OriP-based episomal plasmids do not express any fluorescence marker genes, we co-transfected an additional plasmid of similar size expressing GFP in order to analyze the transfection efficiencies. Our results showed that the 4-in-1 CoMiP (9.4 kb) vector had the highest transfection efficiency (21.45 ± 9.4%) as well as the best cell survival after the transfection compared to the other 2 techniques (Fig. 1A) and Supplementary Fig. 1. The minicircle transfection efficiency (7.6 kb; 15.05 ± 6.86%) analyzed using FACS was comparable to the EBNA/OriP episomal plasmids (~12 kb; 16.35 ± 5.87%), but we observed an increased level of cell death in the minicircle-transfected cells (Fig. 1B) and Supplementary Figs. 1 and 2. One potential reason for this might be residual chromosomal bacterial DNA after the minicircle preparation^12^. Furthermore, by comparing the level of the transgene expression among the independent constructs, we were able to show that the 4-in-1 CoMiP plasmid had the highest Oct4 protein expression level among the reprogramming transgenes (1.6x higher than minicircle and 2.3x higher than regular plasmids) (Supplementary Fig. 3A).

### Single transfection of the 4-in-1 CoMiP plasmid is sufficient to induce pluripotency

**Figure 2:**
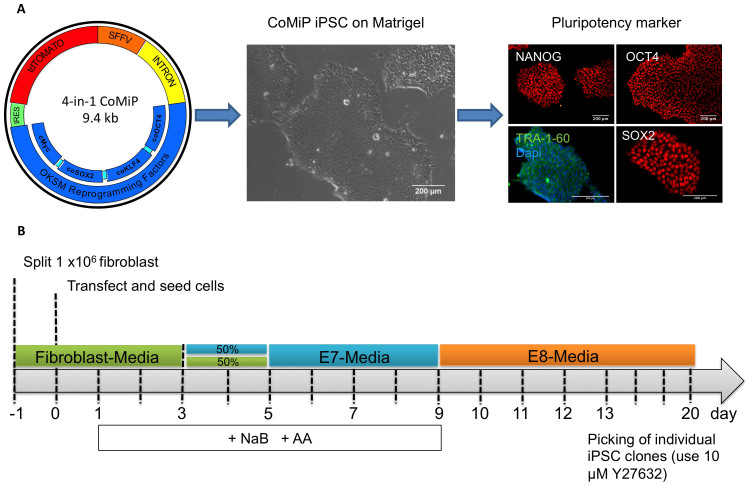
Anticipated workflow and result using the 4-in-1 CoMiP reprogramming vector. (A) Representative brightfield and fluorescent images demonstrating the pluripotent phenotype of the 4-in-1 CoMiP-derived human iPSCs. (B) Detailed timeline shows the media requirements and chemical treatments used for the reprogramming of human fibroblasts, as well as the time frame in which the first iPSCs are ready for further expansion through individual picking.

Initially, our goal here was to optimize our minicircle reprogramming technique^6^. Therefore, we took advantage of a codon-optimized expression cassette of the four canonical reprogramming factors OCT4, KLF4, SOX2, and c-MYC (OKSM) described previously, and combined this cassette with shRNA targeting p53^18^. Using this codon-optimized minicircle (CoMiC) construct, we were able to reprogram adult human fibroblasts via a single nucleofection event^12^. However, the minicircle production process is difficult and time consuming, and the overall reprogramming efficiency was below 0.005%. To overcome these limitations, we used our recently developed expression vector system called mini-intronic plasmid (MiP)^14^. The MiP vector system prevents transgene silencing, which is observed with regular plasmids, by using an engineered intron sequence within the eukaryotic expression cassette. This intronic sequence contains the essential elements required for the bacterial replication and selection of the MiP plasmid (Supplementary Fig. 4). Moreover, we found that the inclusion of the intron into the eukaryotic expression cassette significantly increased the transgene expression for MIP compared to canonical plasmids (2.3-fold lower protein expression) or minicircle (1.6-fold lower protein expression) constructs (Supplementary Fig. 3)^14^. Accordingly, we introduced the codon-optimized reprogramming cassette OKSM together with a tdTomato construct and the p53 shRNA into the MiP backbone vector. Using this 4-in-1 codon-optimized mini-intronic plasmid (4-in-1 CoMiP), we were able to reprogram human adult fibroblasts in less than 2 weeks under feeder-free conditions using chemically-defined media (E7, E8) (Fig. 2). Nearly all emerging iPSC clones were true iPSC colonies and not intermediate “pre-iPSCs,” which randomly differentiated during the reprogramming process^19^. After starting the reprogramming experiment with one million human fibroblasts, a single nucleofection was sufficient to derive approximately 250 iPSC colonies (0.025 ± 0.005%).

### CoMiP plasmid is more efficient than the minicircle or EBNA/OriP episomal plasmid

**Figure 3:**
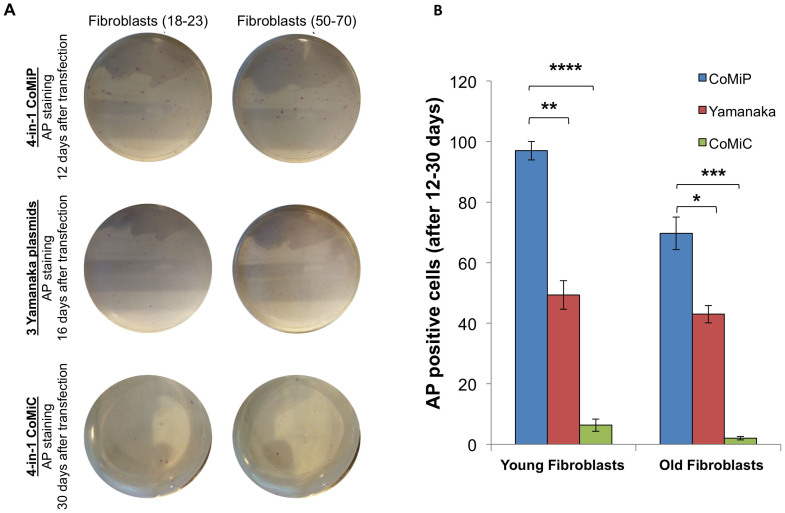
Enhanced induction of pluripotency in human fibroblast using the 4-in-1 CoMiP vector. (A) Alkaline phosphatase (AP) staining revealed faster and superior reprogramming efficiency of the 4-in-1 CoMiP plasmid compared to the 3 Yamanaka episomal plasmids in younger and older human subjects within the first 20 days. (B) The chart summarizes the quantification of the AP-positive iPSC colonies observed in panel (A). Statistical significance was analyzed using the Student's t-test and expressed as a P-value. *P < 0.05; **P < 0.01; ***P < 0.001; ****P < 0.0001; ns, not significant.

We next compared the reprogramming efficiency of our newly developed 4-in-1 CoMiP vector against the minicircle and the Yamanaka EBNA/OriP-based episomal plasmids (Fig. 3). From each reprogramming method, we established at least 5 different iPSC clones and confirmed the pluripotent phenotype of those cells via immunostaining and teratoma assays (Supplementary Fig. 5). To compare the different reprogramming methods, we transfected 1 × 10^6^ fibroblasts from either younger (18–23 years old; n = 5) or older (50–70 years old; n = 5) human subjects with 12 μg total DNA of the different reprogramming plasmids. Afterwards, we plated 5 × 10^5^ of the transfected cells onto a Matrigel-coated plate and changed the media every other day. After 12 days, a significant number of alkaline phosphatase (AP) positive colonies (97 ± 6, per 500,000 transfected cells) appeared in the culture transfected with the 4-in-1 CoMiP vector (Fig. 3A). By contrast, fewer number of AP-positive colonies were evident in the culture transfected with the Yamanaka EBNA/OriP episomal reprogramming plasmids after 16 days (49 ± 9, per 500,000 transfected cells) (Fig. 3A). A comparison of these two different techniques and time points shows a greater than two-fold superior reprogramming efficiency for the 4-in-1 CoMiP vector (Fig. 3B). Moreover, it appears that the higher expression rates of the 4-in-1 CoMiP transgenes led to an early establishment of the pluripotent state within the transfected cells. As shown before, the reprogramming efficiency of the minicircle technique was low for human fibroblasts and highly variable in each individual experiment, resulting in 3–10 potential iPSC clones per transfection (one million cells, below 0.005%) (Figs. 3A, 3B)^6^. Interestingly, we were able to reprogram human fibroblasts using the 4-in-1 CoMiP vector with a single Lipofectamine LTX-mediated transfection, albeit with significantly lower efficiency (0.002%) (Supplementary Fig. 6). By contrast, we could not derive any iPSC clones performing a single lipofection with either the minicircle or the 3 individual Yamanaka reprogramming vectors (Supplementary Fig. 6).

### Induction of pluripotency under chemically-defined conditions and the influence of the innate immune response on the reprogramming efficiency

Because iPSCs or iPSC-derived specialized cell types are potentially relevant for clinical application, it is important to see if we could reprogram human fibroblasts under chemically-defined culture conditions. Therefore, we compared the reprogramming efficiencies of 4-in-1 CoMiP-transfected human fibroblasts initially cultivated in fibroblast media with cells grown under chemically defined conditions (E8 medium plus hydrocortisone on the defined matrix Synthemax)^20^. Although the use of fully chemically-defined conditions reduced the overall number of AP-positive colonies (by at least 50%), we were able to obtain more than 50 colonies by transfecting 250,000 fibroblasts (Supplementary Fig. 7A). Recently, two publications described a significant effect of the innate immune response on reprogramming efficiency by activating either the Toll-like receptor 3 pathway using Polyinosinic-polycytidylic acid (Poly I:C) or by suppressing interferon-induced apoptosis with the Vaccinia virus protein B18R^10^,^21^. These studies utilized either protein (Poly I:C) or mRNA (B18R) reprogramming approaches. Because cytosolic DNA also triggers the innate immune response^22^, we therefore analyzed the 4-in-1 CoMiP-mediated reprogramming efficiency after manipulating the immune response by either Poly I:C or B18R. Our experiments showed a slightly reduced number of AP-positive colonies following activation of the innate immune response by Poly I:C, and a slightly increased number of AP-positive colonies following inhibition of the innate immune response by B18R. However, neither activation nor the inhibition of the innate immune response significantly influenced the CoMiP reprogramming efficiency (Supplementary Fig. 7B).

### CoMiP-derived iPSCs are pluripotent and similar to human ESCs

**Figure 4:**
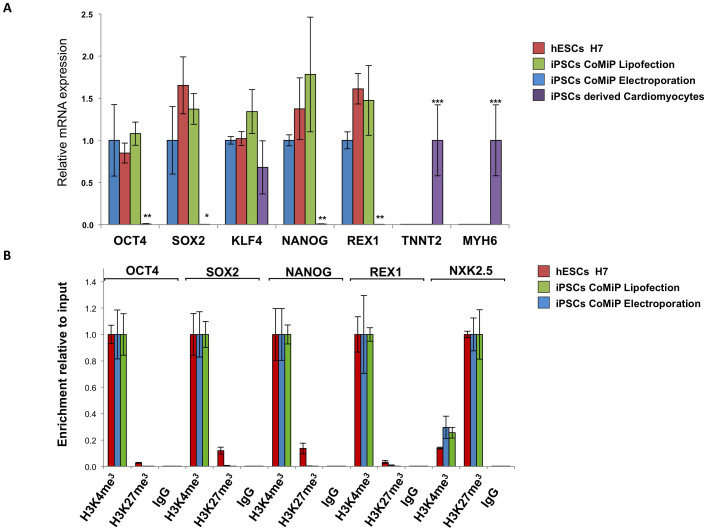
CoMiP-derived iPSCs show a similar gene expression as seen in the hESC line H7. (A–B) 4-in-1 CoMiP-derived iPSCs generated by either electroporation or lipofection showed a similar gene expression and promoter methylation patterns as those observed in the standard human ESC line H7. The 4-in-1 CoMiP-derived iPSCs were negative for the expression of cardiac specific markers such as TNNT2 and MYH6, which were used as a negative control. Statistical significance was analyzed using the student's t-test and expressed as a P-value. *P < 0.05; **P < 0.01; ***P < 0.001; ****P < 0.0001; ns, not significant.

**Figure 5:**
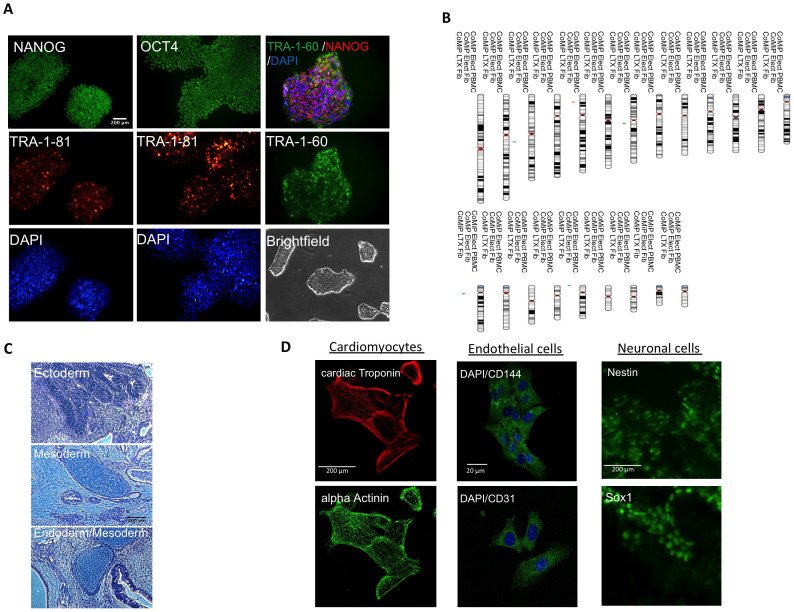
Confirmation of pluripotency of the 4-in-1 CoMiP-derived iPSCs. (A) Immunofluorescence staining on the pluripotency markers NANOG, OCT4, TRA-1-60, and TRA-1-81. DAPI and brightfield images are also shown. (B) 4-in-1 CoMiP-derived iPSCs showed a normal karyotype and (C) from formed *in vivo* teratomas consisting of ectoderm, mesoderm, and endoderm lineages on H&E staining. (D) Directed *in vitro* differentiation into cardiomyocytes, endothelial cells, and neuronal cell using specific monolayer differentiation protocols further confirmed the pluripotent nature of the 4-in-1 CoMiP-derived iPSCs.

We next established multiple 4-in-1 CoMiP-derived iPSC lines using either lipofection or electroporation and confirmed their pluripotency. iPSCs demonstrate the expression of the key pluripotency genes OCT4, NANOG, SOX2, and TRA-1-81, whereas cardiac genes were not expressed compared to differentiated iPSC-derived cardiomyocytes (Fig. 4A). Furthermore chromatin immunoprecipitation (ChiP) followed by qRT-PCR demonstrated that the newly established iPSCs had similar epigenetic pattern as the human ESC line H7 (Fig. 4B). Promoters of pluripotency genes (OCT4, SOX2, NANOG, and REX1) exhibited histone methylation patterns associated with active gene transcription (H3K4me3), whereas promoter regions of lineage specific genes (NKX2.5) exhibited repressive methylation patterns (H4K27me3). All 4-in-1 CoMiP-derived iPSCs showed pluripotent immunostaining and normal karyotype, and were able to differentiate *in vivo* into all three germ layers (Fig. 5A–C). Using a small molecule-based monolayer differentiation protocol, we were also able to differentiate the 4-in-1 CoMiP-derived iPSCs effectively *in vitro* into cardiomyocytes, endothelial cells, and neurons (Fig. 5D)^23^,^24^. A specific PCR assay using primers targeting a 2A-linked junction region between the codon optimized OCT4 and KLF4 regions, and a separate Southern blot analysis using a portion of the tdTomato gene as a probe, confirmed that 4-in-1 CoMiP-derived iPSCs are mainly integration-free (Supplementary Fig. 8). However, some of the CoMiP-derived iPSCs showed traces of plasmid integration or episomal persistence until passage 15 (data not shown). The rate of genomic integration of the 4-in-1 CoMiP plasmid was comparable to the integration rate reported for other reprogramming plasmids^25^. Interestingly, we noticed that the PCR-based screening method for integration is not sufficient. With this method, it is not possible to distinguish among residual episomal persistence, partial plasmid integration, and complete plasmid integration. Therefore, we recommend using the PCR primer as a pre-screening method followed by verification by Southern blot. This combined approach enabled us to identify some iPSCs that showed no sign of integration using PCR primer but were positive for integration in a Southern blot experiment (Supplementary Figs. 8A and 8B).

### Induction of pluripotency in other human somatic cell types and in mouse fibroblasts

**Figure 6:**
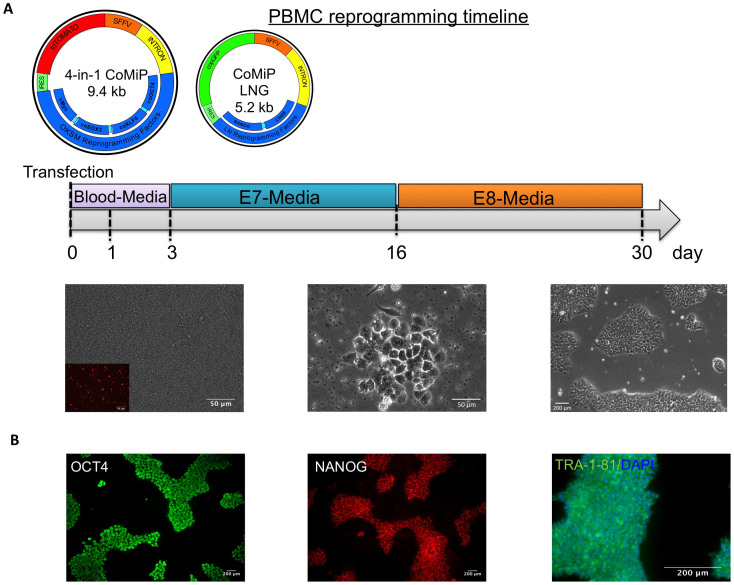
Human peripheral blood mononuclear cells (PBMCs) were successfully reprogrammed using the 4-in-1 CoMiP vector in combination with a Yamanaka vector co-expressing human c-Myc, LIN28, and NANOG. A single transfection of 2 × 10^6^ PBMCs and subsequent cultivation in blood media and chemical defined media was sufficient to generate multiple iPSC colonies. Representative brightfield and fluorescent pictures exemplified the expected outcome of normal PBMC reprogramming experiment. A robust transfection efficiency (tdTomato expression in PBMCs observed 2 days after the electroporation) and optimal culture condition supporting the initial proliferation of the PBMCs are crucial for a successful experimental outcome.

For purposes of clinical application, it is easier to obtain cells for reprogramming via phlebotomy than skin biopsy. For this reason, we next attempted to reprogram freshly isolated peripheral blood mononuclear cells (PBMCs) using the 4-in-1 CoMiP plasmid. Previous studies already described the successful reprogramming of PBMCs with different episomal vectors expressing the canonical reprogramming factors OCT4, KLF4, SOX2, and c-MYC, either together with NANOG and LIN28 or in different combinations^8^,^26^,^27^. Therefore, we transfected 2 × 10^6^ cells with either the 4-in-1 CoMiP plasmid independently or with an additional plasmid expressing LIN28, NANOG, and GFP (CoMiP LNG). As expected, the reprogramming efficiency of the 4-in-1 CoMiP plasmid alone was relatively low (5–7 colonies, 0.00025%), but was up to 3-fold higher when we transfected both plasmids simultaneously (10–17 colonies, 0.00085%). The subsequently isolated iPSC colonies were positive for the expression of the pluripotency markers OCT4, NANOG, and TRA-1-81 (Fig. 6) and demonstrated a normal karyotype (Fig. 5B). Furthermore, we confirmed the pluripotent phenotype of these cells using either the teratoma or the Scorecard assay. The teratoma assay demonstrated the potential of PBMC-derived iPSCs to differentiate *in vivo* into all 3 germ layers (Supplementary Fig. 9A). The Scorecard assay is a real-time PCR-based method comparing pluripotency and differentiation data sets derived with multiple hESC cell^3^. Using this gene expression panel, we also confirmed the pluripotency of our PBMC-derived iPSCs. Furthermore, upon spontaneous differentiation we observed a massive down-regulation of pluripotency-related marker genes and in parallel a strong up-regulation of genes specific for the 3 germ layers (Supplementary Fig. 10A). Based on a previously published robust data set, the Scorecard assay could predict the differentiation propensity of the newly derived iPSCs compared to standardized hESCs^3^. According to those results, our PBMC-derived iPSC clones 1 and 2 have a comparable endoderm and an increased mesoderm and ectodermal differentiation propensity (Supplementary Fig. 10B).

Other clinically relevant and easily obtainable cell types for deriving patient-specific iPSCs include keratinocytes and renal-derived epithelial cells^28^,^29^. Therefore, we also tested our new reprogramming plasmid on these cell types. Similar to what was seen with fibroblasts, a single transfection was enough to reprogram these cells, although with a much lower efficiency in the case of the keratinocytes (5–7 colonies, 0.00025%; Supplementary Fig 11). Interestingly, we observed an accelerated reprogramming time course when transfecting renal epithelial cells obtained from clean catch urine sample, with early iPSCs appearing only 3–5 days after transfection (80–100 colonies, 0.008%; data not shown). Finally, we were also able to derive mouse iPSC colonies from tail fibroblasts of C57Bl6 mice with our 4-in-1 CoMiP construct. This might be of special interest because EBV plasmid-based reprogramming vectors cannot replicate in murine cells, which reduces their reprogramming efficiency^30^. Teratoma assays and immunostaining experiments confirmed the pluripotency of these mouse iPSCs^31^ (Supplementary Fig. 12). A schematic diagram of overall study design and summary of results is shown in Supplementary Fig. 13.

## Discussion

When Shinya Yamanaka and colleagues described the first successful induction of pluripotency in somatic cells in 2006, their reprogramming technique utilized retroviruses that integrated into the host cell genome^5^. Over time, several non-integrating reprogramming techniques based on protein expression, mRNA, or DNA transfection, as well as non-integrating viruses, have been developed^25^. However, most of these alternative techniques are either laborious or relatively expensive. Therefore, the initial aim of our study was to simplify and optimize our previously described minicircle reprogramming technique^6^. To reduce the number of transfections required for the derivation of iPSCs, we introduced a codon-optimized reprogramming cassette carrying the factors OCT4, KLF4, SOX2, and c-MYC together with a tdTomato color marker (coOKSM-IRES-tdTomato) in our minicircle backbone. Using this construct, we were able to reprogram human adult fibroblasts by performing only one transient transfection. However, the reprogramming efficiency of the new 4-in-1 codon optimized minicircle construct (4-in-1 CoMiC) was still extremely low (below 0.002%)^12^. Therefore, we decided to evaluate our newly developed transgene expression vector MiP as an alternative shuttle system for the delivery of the reprogramming factors^14^. We introduced the coOKSM-IRES-tdTomato transgene expression cassette into the MiP backbone, creating the 4-in-1 CoMiP vector. We were able to reprogram numerous somatic human somatic cells (fibroblasts, PBMCs, keratinocytes, and renal epithelial cells) and mouse cell lines (C57Bl6) via a single electroporation. Furthermore, when the 4-in-1 CoMiP vector was cotransfected with a second plasmid expressing LIN28 and NANOG, a single transfection was sufficient to reprogram blood-derived erythroblasts.

Compared to other established non-integrating reprogramming methods, the 4-in-1 CoMiP has multiple advantages. It is color-labeled, time-efficient, inexpensive, and easy-to-use, thus providing an attractive alternative for use in regenerative medicine. In contrast to most of the previously published reprogramming plasmids, the CoMiP system is based on a single plasmid, which does not require the EBNA1 and OriP elements of the Epstein-Barr virus (EBV) for its function^9^,^32^. In addition, because of the engineered intronic region of the polycistronic 4-in-1 CoMiP plasmid, the overall size of our construct was significantly reduced, which in turn increased the transfection efficiency in different somatic cell types. Moreover, the smaller size allowed us to include a RFP marker gene to track the transfection efficiency of our target cell lines. We hypothesize that the higher exogenous transgene expression rate mediated by the intronic sequence and the codon optimization of the 4-in-1 CoMiP plasmid are sufficient to reactivate the endogenous circuit of pluripotency within the first few days, whereas more persistent transgene expression would require other EBNA1- and OriP-based episomal vectors.

Interestingly, we observed a significantly reduced time course for the induction of pluripotency using the 4-in-1 CoMiP, which allowed us to select the first iPSC colonies 12 days after the initial transfection. Furthermore, the 4-in-1 CoMiP reprogramming protocol is compatible to fully chemically defined culture conditions using E7 and E8 media, which may prove to be important for future clinical applications of iPSCs. By contrast, the EBNA1 and OriP sequences used in the Yamanaka episomal reprogramming system are derived from the human pathogenic EBV, and thus carry the risk of possibly altering the innate immune response-triggered gene expression of the subsequently derived iPSCs and may limit their clinical applications^6^. Moreover, the generation and/or function of iPSCs derived from patients carrying an acute or latent infection with EBV (a common occurrence) may be affected by virus-induced overexpression of EBNA1, which in turn could lead to a hyper-expression of the exogenous reprogramming genes^33^.

The use of DNA-based reprogramming methods always carries the potential risk of random genomic integration of the transfected DNA into the host cell genome^34^. Thus, the transfection of fewer plasmids (1 plasmid in the case of the 4-in-1 CoMiP vs. 3 Yamanaka episomal plasmids) is a less risky method for generating integration-free iPSCs. Compared to other single reprogramming plasmids described so far, our 4-in-1 CoMiP protocol is more efficient and is highly reproducible in a broad variety of somatic cell types^8^,^32^,^35^,^36^. Keratinocytes, however, were relatively difficult to reprogram, resulting in only a few iPSC colonies, probably due to the change from specialized keratinocyte media into more chemically-defined conditions of the E7 or E8 media. By contrast, the reason for the enhanced reprogramming process of the renal epithelial cells may be their propensity for mesenchymal-to-epithelial transition and their higher expression levels of E-Cadherin^37^,^38^,^39^,^40^. Therefore, we believe that the reprogramming efficiencies of the different constructs used in this study also depend on the cell type used for the reprogramming experiment^6^,^11^.

Aside from the aforementioned DNA-based reprogramming methods, there are three other commonly used integration-free techniques for inducing pluripotency: protein addition, mRNA transfection, and Sendai virus infection. The disadvantages of the protein reprogramming technique are that it is expensive, inefficient, and slow^21^,^41^. Using modified mRNA for the induction of pluripotency is an elegant and unique approach that guarantees derivation of integration-free iPSCs without any further screening experiments. However, this method is laborious and requires a repetitive series of daily mRNA transfections for up to 14 days and the pre-treatment of the initial cell types with the expensive interferon alpha antagonist B18R^42^. This molecule is crucial for enhancing cell survival during the series of mRNA transfections and for efficient reprogramming^10^. Another limitation of this technique is its dependency on the feeder cell and conditioned media, which brings an extra risk of potentially transmitting undetected human pathogens^43^. A recent publication addressed one of the aforementioned disadvantages of mRNA based reprogramming. Using synthetic, self-replicative RNA, Yoshioka and colleagues showed that a single mRNA transfection was sufficient to derive iPSCs from newborn or adult human fibroblasts^44^. However, further validation is required to establish how robust and reproducible this particular method is. By comparison, the Sendai virus is perhaps the most efficient integration-free reprogramming method currently available. However, it is also the most expensive method, and requires more stringent biosafety containment measures and a separate tissue culture room. The persistence of residual viral material requires an extended period of tissue culture time (10 to 20 passages) to establish virus-free iPSC lines for further downstream analysis and differentiation experiments^25^. Finally, another recently published approach used small-molecule compounds to reprogram mouse somatic cells^45^. However, the efficiency of this technique is also quite low, and the study must be reproduced using human somatic cells in order to be of broader clinical interest.

In summary, the 4-in-1 CoMiP is an effective alternative reprogramming method for deriving integration-free iPSCs from various donor tissue sources, including PBMCs under chemically defined conditions without using animal-derived products. Compared to other DNA-based reprogramming methods like the minicircle or the Yamanaka three individual plasmids system, the CoMiP construct is faster and less expensive for inducing pluripotency in a somatic cell type (Supplementary Fig. 13). Furthermore, the cost effectiveness of our new reprograming technique should be emphasized because of the current impetus for major research institutions to generate large-scale iPSC banks. For example, the estimated cost for the consumables per derived iPSC line is ~$80 for 4-in-1 CoMiP versus $500 for Sendai virus. Hence this CoMiP construct will allow novice and inexperienced researchers alike to easily obtain *bona fide* iPSCs within 14 days. Aside from being the fastest reprogramming method, the 4-in-1 CoMiP plasmid does not result in intermediate iPSCs and, due to its color label, the entire reprogramming process is easy to monitor. With all these superior qualities, we believe that this new technology is of special interest given the great potential of iPSCs in regenerative medicine.

## Methods

### Construction of the 4-in-1 CoMiP construct

The 4-in-1 CoMiP construct was constructed from the 4-in-1 CoMiC vector. The 1.4 kb OIPR intron carrying pUC origin and RNA-OUT selectable marker was PCR-amplified from the MIP parental plasmid by using forward primer “5′ATTGGGATCTTCACACAGCA3′” and reverse primer “5′TTAGCTAGTCAGCTAGTGGAC3′”. *AgeI* sites were incorporated into the 5′ end of both primers. The PCR product was digested with *AgeI* restriction enzyme and then ligated with *AgeI*-digested 4-in-1 CoMiC. The *AgeI* cut 4-in-1 CoMiC once at the 3′ end of SFFV promoter. The ligation was selected on LB solid media with 6% sucrose (the condition for RNA-OUT selection). The positive colonies were then selected for sequencing to determine the insertion orientation in the resultant 4-in-1 CoMiP plasmid. The U6-driven p53 expression cassette was PCR-amplified from the pCXLE-hOCT3/4-shp53-F vector by using the forward primer “5′GACGCCGCCATCTCTAGG3′” and reverse primer “5′CCCGGGCTGCAGGAATTC3′”. *SpeI* sites were incorporated into the 5′ end of both primers. The *SpeI-*digested U6-p53 PCR product was then ligated with *XbaI* digested 4-in-1 CoMiP. *XbaI* cut 4-in-1 CoMiP once in the short (36 bp) spacer backbone region. The ligation was then selected on LB solid media with 6% sucrose and the positive colonies were selected for sequencing. However, only the backward insertion was able to grow in this experiment design. This provided the final version of 4-in-1 CoMiP vector.

### Production of minicircle, episomal plasmid, and CoMiP vectors

The minicircle plasmids were produced as described previously^6^,^12^. The episomal reprogramming plasmids used for this study were purified using the QIAGEN Plasmid Maxi Kit (QIAGEN, USA). The 4-in-1 CoMiP construct was transformed into NTC4862 DH5α competent cells and then plated on 6% sucrose solid media (see Nature Technology's NTC vector User's Manual) and propagated at 30°C for 24–48 hours. Individual colonies were picked and miniprep was performed (QIAprep Spin Miniprep Kit, USA). Thereafter, a 100 μl aliquot of the 4-in-1 CoMiP bacterial culture was inoculated with 100 ml of 6% sucrose liquid media and incubated for 16–18 hr at 37°C. This culture was then used to isolate the 4-in-1 CoMiP vector by using the QIAGEN Plasmid MAXI Kit.

### Reprogramming using 4-in-1 CoMiC, 4-in-1 CoMiP, or episomal plasmids

Reprogramming using the minicircle technique was performed as previously described with an optimized minicircle backbone (4-in-1 CoMiC) and some changes in the reprogramming procedure^11^. To increase cell survival after electroporation, the minicircle DNA was purified using Zymoclean Gel DNA Recovery Kit (Zymo Research, USA). The DNA of either the 4-in-1 CoMiP plasmid or the 3 individual Yamanaka episomal plasmids was isolated according to the QIAGEN Plasmid Plus Maxi Kit instructions. The following paragraph describes the fibroblast transfection procedure, which was identical for all reprogramming plasmids used in this study. One day before the reprogramming experiment, 1 × 10^6^ human fibroblasts were plated onto a 10- cm dish. On the ensuing day, the cells were trypsinized and electroporated with 12 μg DNA of the reprogramming plasmids (12 μg of 4-in-1 minicircle CoMiC or 4-in-1 CoMiP, 4 μg each of the individual Yamanaka episomal plasmids) using the Invitrogen Neon system (1600 volt, 10 ms, and 3 pulses, transfection efficiency should be more than 50%). Subsequently, these cells were equally distributed onto one or two Matrigel-coated plates (5.5 × 10^5^ cells) in fibroblast media. On the following morning (day 1), the media was changed to fibroblast media with the addition of 0.2 mM sodium butyrate plus 50 μg/mL ascorbic acid. On day 3, the media was changed to Essential 7 media supplemented with 0.2 mM sodium butyrate and 50 μg/mL ascorbic acid. Between day 6 (4-in-1 CoMiP) and day 20 (4-in-1 minicircle CoMiC, Yamanaka episomal plasmids) the first iPSC-like colonies appeared, after which we switched the culture conditions to Essential 8 medium and hypoxic conditions. Around days 12–30, the iPSC colonies were large enough for manual selection under the microscope. In general, we selected 6 individual iPSC clones using the Vitrolife stem cell cutting tool with a 10 μL tip, and transferred them into 6 different wells of a Matrigel-coated plate with Essential 8 media in the presence of 10 μM ROCK inhibitor (Y-27632). After 5–7 days, the individual colonies became big enough to dissociate into single cells using 0.5 mL TrypLE for 8 minutes. Thereafter, we washed the cells once and passaged them into one well of a Matrigel-coated 6-well plate containing 2 mL Essential 8 media and 10 μM ROCK inhibitor (Y-27632).

### Statistical Analysis

Data shown are presented as mean ± standard error mean (SEM) of four or more independent experiments. Differences are considered statistically significant at P < 0.05, and assessed using the Student's t test (for paired samples).

## Author Contributions

S.D. conceived, performed, and interpreted the experiments and wrote the manuscript; J.L. developed the MIP vector system and cloned the 4-in-1 CoMiP vector; N.G.K. performed the reprogramming of mice fibroblast and the cell injections; J.C.L. did the real-time PCR and chromatin immunoprecipitation experiments; V.T. helped with the revision and did the scorecard analysis; J.M.C. helped with the episomal plasmid reprogramming experiments; P.W.B. helped with the CoMiP reprogramming, A.D.E. and A.S. performed the cardiomyocyte differentiation and immunofluorescence analysis; M.A.K. helped with the experimental design and manuscript writing; and J.C.W. provided experimental advice, manuscript writing, and funding support.

## Supplementary Material

Supplementary Information Supplemental Materials and methods and figures
