# Supplementary material for: Novel codon-optimized mini-intronic plasmid for efficient, inexpensive, and xeno-free induction of pluripotency
Source: Sci Rep. 2015 Jan 28;5:8081. doi: 10.1038/srep08081 (PMC4308704; doi:10.1038/srep08081)
Supplement: Supplementary Information — Supplemental Materials and methods and figures [file srep08081-s1.pdf]

SUPPLEMENTAL MATERIALS

Novel codon-optimized mini-intronic plasmid for efficient, inexpensive, and xeno-free induction of pluripotency

Sebastian Diecke[1,2,3,5,6], Jiamiao Lu[4], Jaecheol Lee[1,2,3], Vittavat Termglinchan[1,2,3] , Nigel G. Kooreman[1,2,3], Paul W. Burridge[1,2,3], Antje D. Ebert[1,2,3], Jared M. Churko[1,2,3], Arun Sharma[1,2,3], Mark A. Kay[4], Joseph C. Wu[1,2,3]

[1]Institute for Stem Cell Biology and Regenerative Medicine; [2]Stanford Cardiovascular Institute; [3]Department of Medicine, Division of Cardiology; [4]Departments of Pediatrics and Genetics; Stanford University School of Medicine, Stanford, California 94305, USA;
[5]Max Delbrück Center, Robert-Rössle Strasse 10, 13125 Berlin, Germany; [6]Berlin Institute of Health, Luisenstraße 56, 10117 Berlin, Germany

SUPPLEMENTARY METHODS

Screening for potential plasmid integration by Southern blot or PCR. Genomic DNA was extracted from CoMiP-transfected cell line, lentivirus-infected control cell line, and negative control fibroblast cell line. Twenty µg of genomic DNA from each sample were digested with SpeI overnight at 37˚C. SpeI cut the CoMiP expression cassette once and linearized the vector. Digested DNA samples were separated by electrophoresis on a 1% agarose gel and blotted onto a nitrocellulose membrane. 0.1 ng SpeI-digested or non-digested CoMiP vector DNA was incorporated into the same Southern gel as linearized or non-linearized non-integrating control. Twenty µg SpeI-digested genomic DNA of negative control fibroblasts were mixed with one CoMiP vector linearized non-integrating control sample to imitate genomic DNA extracted from cell lines. The Southern blot membrane was hybridized with [P-32] dCTP-labeled 702 bp tdTomato probe (PCR product by using forward primer "5'GTGAGCAAGGGCGAGGAGG3'" and reverse primer "5'CTTGTACAGCTCGTCCATGC3'") overnight at 65°C. CoMiP signals were detected through phosphoimaging. The PCR screening was performed by using the standard Taq polymerase protocol and primers summarized in Supplementary Table 1.

Analysis of protein expression differences by Western blot. Cells were harvested in RIPA lysis buffer (Sigma) supplemented with protease (Complete Mini; Roche) and phosphatase inhibitors (Phosphatase Inhibitor Cocktail I and II; Sigma). Five to twenty µg of total protein were separated by SDS-PAGE and transferred to polyvinylidene fluoride membranes following the protocol of the manufacturer (Invitrogen XCell SureLock™ Mini Cell Electrophoresis and Wet Blot System). Membranes were incubated overnight with the primary antibodies and with appropriate peroxidase-conjugated secondary antibodies for 45 min. Chemiluminescence

(PerkinElmer) reaction was carried out. Primary antibodies used were against OCT4 (R&D Systems, AF1759) and α-tubulin (Sigma). Secondary antibodies were anti-goat and anti-mouse horseradish peroxidase (Jackson Laboratories).

Immunofluorescence of human iPSCs. Human iPSCs ($1x10^4$ cells/chamber) derived with the different methods were seeded on Matrigel coated 8-chamber slides in a final volume of 200 μL of E8 medium. After 24-72 hr, cells were washed 3X with PBS and fixed for 20 min at RT using 100 μL 4% paraformaldehyde (PFA) in PBS. Thereafter, cells were washed with 200 μL PBS (3X) and incubated for 15 min at RT with 100 μL of permeabilization solution (0.2% Triton X-100 in PBS). Next, cells were washed twice with PBS and incubated with blocking solution (2% BSA or 4% goat serum or 2% FBS in PBS), for 1 hr at RT or overnight at 4ºC. After another wash step (PBS), cells were covered overnight at 4°C with primary antibody diluted appropriately in blocking solution (100 μL per chamber). The following day, slides were washed 4X for 10 min using 1% goat serum, 0.1% Tween-20 in PBS. Afterwards, cells were incubated with the appropriate secondary antibody diluted in blocking solution, for 1 hr at RT (100 μL per chamber). After a final wash step (4X, 1% goat Serum, 0.1% Tween-20 in PBS, for 10 min), chambers were removed and cells were incubated with a small amount of Vectashield mounting medium with DAPI. Afterwards, cells were covered and sealed using nail varnish.

Immunofluorescence of human iPSC-derived cardiomyocytes. iPSC-CMs were passaged onto Matrigel-coated coverslips and immunohistochemistry was performed as described before (Lan et al., Cell Stem Cell 2013). Samples were analyzed using a LSM 510 Meta inverted confocal microscope (Carl Zeiss) and ZEN software (Carl Zeiss).

Karyotype analysis of human iPSCs. The iPSC lines were detached from the culture plate using Accutase for 8 min and centrifuged at 300 g for 3 min. iPSC pellets were resuspended in 200 μL of PBS and DNA was isolated using the DNeasy blood and tissue kit (Qiagen, Valencia, CA). DNA was submitted to the Stanford Functional Genomics Facility for SNP karyotyping using the HuCytoSNP-12 chip (Illumina). CNV and SNP visualization were performed using KaryoStudio v1.4 (Illumina).

RNA extraction and quantitative real-time PCR. Total RNA was isolated using RNeasy Plus Mini Kit (Qiagen 74134). The reverse transcriptase PCR was performed using the iScript™ cDNA Synthesis Kit (Bio-rad 170-8891). The quantitative real-time PCR was performed using the iQ SYBR Green Supermix (Bio-rad 170-8882) and a CFX96™ real-time PCR detector (Bio-Rad). The relative mRNA levels were normalized to the values of 18S mRNA for each reaction. Primer information shown in Supplementary Table 2.

Chromatin immunoprecipitation assays. The antibodies used in ChIP assays were 39159 for H3 tri-methylation lysine4 (Active Motif), 39155 for H3 tri-methylation lysine27 (Active Motif), and sc-2027 for control IgG (Santa Cruz Biotechnology). Antibodies were incubated with Dynabeads (Invitrogen, 10003D) for 12 hr at 4°C. The main fraction of the crosslinked and sheared chromatin was incubated with the antibody-conjugated Dynabeads and a small aliquot of the chromatin was used as an input control. After an overnight incubation at 4°C, the incubated beads were rinsed with sonication buffer (50 mM Hepes pH 7.9, 140 mM NaCl, 1 mM EDTA, 1% Triton X-100, 0.1% Na-deoxycholate, 0.1% SDS, 0.5 mM PMSF), high salt buffer (50 mM Hepes pH 7.9, 500 mM NaCl, 1 mM EDTA, 1% Triton X-100, 0.1% Na-deoxycholate, 0.1%

SDS, 0.5 mM PMSF), and LiCl buffer (20 mM Tris, pH 8.0, 1 mM EDTA, 250 mM LiCl, 0.5% NP-40, 0.5% Na-deoxycholate, 0.5 mM PMSF). The washed beads were incubated with an elution buffer (50 mM Tris, pH 8.0, 1 mM EDTA, 1% SDS, 50 mM $NaHCO_3$) for 1 hr at 65°C and then de-crosslinked overnight with 5M NaCl at 65°C. The immunoprecipitated DNA was treated with Rnase A and Proteinase K, and purified by ChIP DNA clean and concentrator (Zymo Research D5205). The immunoprecipitated and input DNA was analyzed by quantitative real-time PCR. Primer information is shown in Supplementary Table 3.

Fluorescent activated cell sorting (FACS). Transfected fibroblasts were detached using TrypLE (Life Technologies) and stained when indicated with propidium iodide (PI) to determine the percentage of viable cells. Samples were analyzed using a LSR Fortessa Analyzer and BD FACSDiva, as well as FlowJo softwares.

Scorecard pluripotency and differentiation panel. iPSC or iPSC-derived embryoid bodies (10 days old) were harvested either using Trizol or RNeasy Mini Kit (QIAGEN). Subsequently, samples were further processed as described in TaqMan® hPSC Scorecard™ Panel User Guide (Life Technologies). Samples were prepared in 384-well plate and analyzed by 7900HT System.

SUPPLEMENTARY FIGURE LEGENDS

Supplementary Figure 1. Comparison of the transfection efficiencies using different reprogramming plasmids. **(A-B)** FACS blot showing the comparable transfection efficiencies of CoMiP, CoMiC, and 3 Yamanaka factors.

Supplementary Figure 2. Cell viability after transfection of the individual reprogramming plasmids. **(A-C)** Using propidium iodide staining followed by FACS analysis, we observed the highest cell survival after transfecting with the CoMiP reprogramming plasmid.

Supplementary Figure 3. Analysis of the transient transgene expression levels in HEK 293 cells produced by the different constructs used in this study (minicircle, CoMiP, and regular Plasmid). **(A)** Codon-optimized reprogramming cassette OKSM cloned into minicircle, mini-intronic plasmid (MiP), or regular plasmid backbone were transfected into HEK 293 cells. Western blot illustrates that the level of protein expression is dependent on the plasmid backbone. All constructs had the same SFFV promoter sequence driving the OKSM expression. **(B)** Comparison of the transgene expression efficiency mediated by different constructs (Yamanaka episomal plasmids, pCXL, and the 4-in-1 CoMiP) showed superior OCT4 protein expression (Western blot) levels by the CoMiP construct.

Supplementary Figure 4. Schematic overview of the 4-in-1 codon-optimized reprogramming plasmid (CoMiP). The plasmid-defining intronic sequence contains the antibiotic-free bacterial selection cassette. The strong spleen focus forming virus (SFFV) promoter drives the expression

of the reprogramming factors that are linked to a fluorescent marker (tdTomato) through an internal ribosomal entry site (IRES) site. Addition of a p53 shRNA further increased the reprogramming efficiency.

Supplementary Figure 5. (A) Immunofluorescence staining (TRA-1-60, OCT4 and nuclear DAPI staining) and **(B)** *in vivo* teratoma differentiation assays confirmed pluripotency of the iPSCs derived with the 3 individual Yamanaka episomal plasmids pCXLE-hOCT3/4-shRNA p53, pCXLE-hl-Myc/LIN28, and pCXLE-hSOX2/KLF4. **(C)** The pluripotent identity of the iPSCs derived with the 4-in-1 minicircle (CoMiC) was confirmed by Brightfield picture Immunofluorescence staining (OCT4 and NANOG) and **(D)** *in vivo* teratoma differentiation assays.

Supplementary Figure 6. Comparison of the three different plasmid-based reprogramming strategies on human fibroblasts. CoMiC, CoMiP, and episomal plasmids were transiently introduced into fibroblasts of **(A)** young (18-23 years) versus **(B)** old (50-70 years) human subjects using lipofection. Using the 4-in-1 CoMiP plasmid, we were able to derive 3-10 iPSC colonies after transfecting $2x10^5$ cells in a single attempt, whereas we could not generate any iPSCs by using a single-pass lipofection with the minicircle or episomal plasmid. Representative pictures showing different AP positive cell colonies (magnification) and a proliferating pre-iPSC clone.

Supplementary Figure 7. Analysis of the reprogramming efficiencies under chemical defined condition and influences of the innate immune response. **(A)** Robust 4-in-1 CoMiP-mediated

reprogramming efficiency under chemical defined conditions using Synthemax and chemical defined media E7. However, reprogramming using fully chemical defined condition reduced the overall number of AP-positive colonies by roughly 50%. **(B)** Manipulating the innate immune response had no statistically significant influence on the 4-in-1 CoMiP-mediated reprogramming efficiency. Activation of the innate immune response by poly I:C slightly reduced the number of AP-positive cells, whereas inhibition of the innate immune response by B18R slightly enhanced the number of AP-positive cells. Statistical significance was analyzed using the student's t-test and expressed as a P-value. *P < 0.05; **P < 0.01; ns, not significant.

Supplementary Figure 8. Reprogramming with 4-in-1 CoMiP plasmid allows derivation of integration-free iPSCs. **(A)** Southern blot experiments were used to screen different 4-in-1 CoMiP-derived iPSCs for potential integrations. Lanes 0 and 1 represent control DNA either from untransfected 4-in-1 CoMiP plasmid DNA (internal control for the Southern blot probe targeting the tdTomato sequence, 0) or from a lentiviral-derived iPSC line (1, integration control). Clones 2, 4, and 6 were integration-free and also free of residual plasmid DNA. Clones 3, 5 and 7 might be free of integration but still had residual CoMiP DNA within the individual clones as circulating episomes. The clone at line 8 shows multiple genomic integrations. **(B)** PCR-based screening method for potential integration using primers targeting the junction region between OCT4 and KLF4 within the 4-in-1 CoMiP plasmid mainly confirms the Southern blot results, albeit with a lower specificity (clones 7 and 8 appear negative).

Supplementary Figure 9. PBMC, keratinocytes, and renal epithelia-derived iPSCs are pluripotent. After injection into nude mice, all of the individual iPSCs generated teratomas representing all 3 germ layers.

Supplementary Figure 10. Pluripotency analysis of the PBMC, keratinocytes and renal epithelia-derived iPSCs using the TaqMan® hPSC Scorecard™ Panel. **(A)** The individual iPSC lines showed overall expression scores of pluripotency genes that were comparable to the reference. After spontaneous differentiation into embryoid bodies, the expression of the self-renewal genes were down-regulated and the expression of genes of the 3 germ layers were up-regulated. **(B)** All cell lines showed a high propensity to differentiate into the 3 germ layers.

Supplementary Figure 11. CoMiP reprogramming of human keratinocytes. **(A)** Timeline shows the expected morphological changes of 4-in-1 CoMiP-transfected and reprogrammed human keratinocytes. **(B)** The expression of different pluripotency related marker genes within the keratinocyte-derived iPSCs was confirmed by immunofluorescence staining for OCT4, NANOG, and TRA-1-81.

Supplementary Figure 12. The pluripotency of the 4-in-1 CoMiP-derived murine iPSC cell lines were confirmed by *in vitro* and *in vivo* experiments. **(A)** Immunofluorescence staining showed the expression of different pluripotency associated marker genes (Nanog, Oct4, and SSEA-1). **(B)** Teratoma assay confirmed the pluripotent potential of murine iPSCs to form ectoderm, endoderm, and mesoderm layers *in vivo*.

Supplementary Figure 13. Summarizing the study design of the comparison of the three reprogramming methods. **(A)** One single nucleofection was sufficient enough to reprogram human adult fibroblast using any one of the three methods. Using the 4-in-1 CoMiP vector system was faster than the episomal or minicircle reprogramming methods without generating intermediate iPSCs (blue cell cluster) but only fully reprogrammed iPSCs (green cell cluster). **(B)** Interestingly, only the 4-in-1 CoMiP vector system was able to induce pluripotency in adult human fibroblasts by using one single lipofection to deliver the reprogramming plasmids. AP, alkaline phosphatase.

SUPPLEMENTARY TABLES

Supplementary Table 1

Primer sequences for integration test:

OCT4-KLF4 Set2	**F**: GGCAGAAGGGCAAGAGAAG
	**R**: CTCCCGCCATCTGTTGTTAG

Supplementary Table 2

Primer sequences for qPCR:

NANOG	**F**: TTCACTGTGTTAGCCAGGATG	**R**: ATGTCTTTTCTAGGCAGGGC
SOX2	**F**:GAGAAGTTTGAGCCCCAGG	**R**: AGAGGCAAACTGGAATCAGG
REX1	**F**: TTCTCCTTTGTTTTACGTTTGGG	**R**: AGATCACACCATTGCACTCC
KFL4	**F**: ACCTACACAAAGAGTTCCCATC	**R**: TGTGTTTACGGTAGTGCCTG
OCT4	**F**: CCATGCATTCAAACTGAGGTG	**R**: CCTTTGTGTTCCCAATTCCTTC
TNNT2	**F**: AGCGGAAAAGTGGGAAGAG	**R**: TCCAAGTTATAGATGCTCTGCC
MYH6	**F**: GGGACAGTGGTAAAAGCAAAG	**R**: ACAAAGTGAGGATGGGTGG
18s	**F**: GAGACTCTGGCATGCTAACTAG	**R**: GGACATCTAAGGGCATCACAG

Supplementary Table 3

Primer sequences for ChIP-qPCR:

NANOG-ChIP	**F**: AGAGAAGGAGAGAGGGTTAAGG
	**R**: GCAGGTGCCACCAAGTTAT
OCT4-ChIP	**F**: CTTGGGTCTGATAAGGGTCAAA
	**R**: CAGGGTCTCTCACATCTCCTA
SOX2-ChIP	**F**: CATGAACGGCTGGAGCAA
	**R**: GCTGGTCATGGAGTTGTACTG
REX1-ChIP	**F**: AAGTGTCTGGCGGTGATTT
	**R**: CTCCAGAGATAAGAGGAGAGGAA
NKX2.5-ChIP	**F**: GTAAGAGCGGCTTGACCTAC
	**R**: CTGACTTTCTACAAATGCTGAGTG

Supplementary Table 4 (Scorecard results)

Scorecard Analysis of iPSC Lines (Fold-change)

Target Name	Category	PBMC Cl1	Keratinocyte	Renal	PBMC Cl2
CDH9	Ectoderm	0,30	0,07	0,18	0,36
COL2A1	Ectoderm	3,43	0,39	1,57	2,18
DMBX1	Ectoderm	0,06	0,09	0,46	0,13
DRD4	Ectoderm	0,08	0,01	0,09	0,06
EN1	Ectoderm	1,51	0,63	7,21	1,38
LMX1A	Ectoderm	0,37	0,12	1,50	0,43
MAP2	Ectoderm	5,65	0,58	0,18	6,06
MYO3B	Ectoderm	2,89	0,61	1,13	2,85
NOS2	Ectoderm	0,15	0,22	0,26	0,11
NR2F1/NR2F2	Ectoderm	0,39	0,04	0,08	0,46
NR2F2	Ectoderm	19,76	0,75	4,93	19,91
OLFM3	Ectoderm	0,46	0,51	0,52	0,48
PAPLN	Ectoderm	0,89	0,23	1,45	0,84
PAX3	Ectoderm	111,11	1,73	4,04	73,80
PAX6	Ectoderm	2,17	0,15	0,27	2,62
POU4F1	Ectoderm	0,64	0,72	1,90	0,07
PRKCA	Ectoderm	0,37	0,28	0,27	0,36
SDC2	Ectoderm	9,79	6,22	16,61	10,69
SOX1	Ectoderm	0,03	0,04	0,07	0,20
TRPM8	Ectoderm	25,24	2,40	1,28	28,11
WNT1	Ectoderm	0,22	0,24	0,47	0,18
ZBTB16	Ectoderm	0,64	0,25	1,27	0,72
AFP	Endoderm	0,01	0,01	0,01	0,00
CABP7	Endoderm	0,00	0,04	0,83	0,00
CDH20	Endoderm	1,04	0,47	0,46	1,00
CLDN1	Endoderm	1,01	0,47	0,56	1,22
CPLX2	Endoderm	0,02	0,05	0,02	0,01
ELAVL3	Endoderm	0,07	0,70	4,88	0,07
EOMES	Endoderm	0,03	0,02	0,06	0,04
FOXA1	Endoderm	0,45	0,14	0,64	0,05
FOXA2	Endoderm	0,00	0,60	0,01	0,00
FOXP2	Endoderm	0,37	0,37	0,42	0,33
GATA4	Endoderm	0,02	0,04	0,24	0,01
GATA6	Endoderm	0,00	0,01	0,03	0,01
HHEX	Endoderm	0,07	0,09	0,48	0,07
HMP19	Endoderm	0,42	1,24	1,74	0,34
HNF1B	Endoderm	0,21	0,03	0,17	0,12
HNF4A	Endoderm	0,00	0,27	0,06	0,00
KLF5	Endoderm	1,39	1,95	3,05	1,30
LEFTY1	Endoderm	0,04	1,07	0,52	0,05

LEFTY2	Endoderm	0,22	0,95	0,60	0,24
NODAL	Endoderm	0,01	0,26	0,27	0,01
PHOX2B	Endoderm	0,13	0,14	0,28	0,11
POU3F3	Endoderm	0,53	0,06	0,67	0,51
PRDM1	Endoderm	0,14	0,03	0,07	0,13
RXRG	Endoderm	1,93	0,20	0,05	1,47
SOX17	Endoderm	0,02	0,17	1,97	0,18
SST	Endoderm	12,84	4,76	1,63	16,16
FGF4	Mesendoderm	0,12	13,55	51,21	0,08
GDF3	Mesendoderm	0,68	11,90	8,75	0,87
NPPB	Mesendoderm	0,02	0,08	0,01	0,09
NR5A2	Mesendoderm	159,74	372,92	532,81	160,16
PTHLH	Mesendoderm	1,58	0,29	0,58	1,41
T	Mesendoderm	0,00	0,00	0,00	0,01
ABCA4	Mesoderm	1,64	1,58	2,80	1,90
ALOX15	Mesoderm	0,12	0,21	0,41	0,15
BMP10	Mesoderm	0,28	0,29	0,58	0,22
CDH5	Mesoderm	54,01	1,98	1,73	54,66
CDX2	Mesoderm	0,32	0,26	0,25	0,26
COLEC10	Mesoderm	0,57	0,48	2,21	0,77
ESM1	Mesoderm	0,50	1,02	1,81	0,66
FCN3	Mesoderm	1,12	1,41	6,03	1,13
FOXF1	Mesoderm	0,91	2,22	5,59	1,47
HAND1	Mesoderm	0,02	0,38	1,31	0,09
HAND2	Mesoderm	1,62	2,85	3,59	0,48
HEY1	Mesoderm	0,82	0,27	0,37	0,79
HOPX	Mesoderm	0,28	2,36	4,67	4,87
IL6ST	Mesoderm	1,17	0,73	1,32	1,11
NKX2-5	Mesoderm	0,87	4,64	1,96	0,38
ODAM	Mesoderm	0,25	2,53	3,29	1,42
PDGFRA	Mesoderm	0,09	1,02	0,74	0,10
PLVAP	Mesoderm	0,89	0,32	0,77	0,81
RGS4	Mesoderm	0,15	1,06	0,45	0,15
SNAI2	Mesoderm	3,40	0,29	0,20	3,79
TBX3	Mesoderm	0,02	0,08	0,20	0,02
TM4SF1	Mesoderm	11,15	0,60	0,21	14,61
CXCL5	Self-renewal	23,67	4,46	5,59	31,89
DNMT3B	Self-renewal	omitted	omitted	omitted	omitted
HESX1	Self-renewal	omitted	omitted	omitted	omitted
IDO1	Self-renewal	0,15	0,30	0,33	0,17
LCK	Self-renewal	1,67	1,66	2,59	1,27
NANOG	Self-renewal	0,49	1,07	1,55	0,40
POU5F1	Self-renewal	0,31	0,70	0,92	0,30
SOX2	Self-renewal	0,53	0,52	1,07	0,69
TRIM22	Self-renewal	0,91	0,37	0,81	1,00

Fold-change legend

fc > 100	Upregulated
10 < fc <= 100	
2 < fc <= 10	
0.5 <= fc <= 2	Comparable
0.1 <= fc < 0.5	
0.01 <= fc < 0.1	
fc < 0.01	Downregulated
omitted	

Scorecard Analysis of Embryoid Bodies (Fold-change)

Experiment Filename				
**Target Name**	**Category**	**PBMC Cl1**	**Renal**	**PBMC Cl2**
CDH9	Ectoderm	0,26	0,02	0,51
COL2A1	Ectoderm	31,89	0,65	21,25
DMBX1	Ectoderm	1,05	0,01	0,74
DRD4	Ectoderm	0,33	0,27	1,39
EN1	Ectoderm	102,37	3,75	5,81
LMX1A	Ectoderm	157,80	0,22	81,69
MAP2	Ectoderm	25,76	0,21	10,09
MYO3B	Ectoderm	21,22	20,75	25,59
NOS2	Ectoderm	0,11	14,22	0,40
NR2F1/NR2F2	Ectoderm	84,81	0,17	133,13
NR2F2	Ectoderm	890,29	1.357,35	872,57
OLFM3	Ectoderm	48,53	0,52	44,44
PAPLN	Ectoderm	0,31	0,46	0,31
PAX3	Ectoderm	672,77	7,25	2.505,62
PAX6	Ectoderm	26,99	0,12	151,11
POU4F1	Ectoderm	39,66	1,16	15,34
PRKCA	Ectoderm	1,52	0,26	0,29
SDC2	Ectoderm	52,75	2,52	10,21
SOX1	Ectoderm	0,94	0,06	1,96
TRPM8	Ectoderm	86,50	1,01	12,72
WNT1	Ectoderm	3.542,76	65,58	3.343,77
ZBTB16	Ectoderm	9,25	0,40	18,89
AFP	Endoderm	18,80	1,46	0,01
CABP7	Endoderm	3,26	0,32	4,00
CDH20	Endoderm	7,75	0,39	53,48
CLDN1	Endoderm	3,39	10,65	7,20
CPLX2	Endoderm	10,33	0,13	1,36
ELAVL3	Endoderm	13,76	0,30	5,42
EOMES	Endoderm	0,03	0,37	0,09
FOXA1	Endoderm	3,22	0,38	0,24

FOXA2	Endoderm	0,81	0,49	0,01
FOXP2	Endoderm	19,19	0,64	10,72
GATA4	Endoderm	0,79	1,13	0,06
GATA6	Endoderm	0,74	2,07	0,35
HHEX	Endoderm	0,89	0,19	0,06
HMP19	Endoderm	50,30	5,74	8,28
HNF1B	Endoderm	5,70	1,22	2,80
HNF4A	Endoderm	2,79	1,13	0,06
KLF5	Endoderm	2,74	15,93	12,36
LEFTY1	Endoderm	0,01	0,03	0,02
LEFTY2	Endoderm	0,23	0,11	0,74
NODAL	Endoderm	0,01	0,06	0,06
PHOX2B	Endoderm	10,11	0,24	7,21
POU3F3	Endoderm	3,03	0,01	1,77
PRDM1	Endoderm	0,42	1,29	0,47
RXRG	Endoderm	94,86	12,02	35,09
SOX17	Endoderm	36,51	13,05	0,26
SST	Endoderm	133,19	32,06	132,01
FGF4	Mesendoderm	0,04	4,87	0,21
GDF3	Mesendoderm	0,07	3,76	0,46
NPPB	Mesendoderm	0,58	3,25	9,02
NR5A2	Mesendoderm	17,90	9,67	98,89
PTHLH	Mesendoderm	120,07	15,16	52,43
T	Mesendoderm	0,00	0,04	0,01
ABCA4	Mesoderm	12,06	30,33	26,86
ALOX15	Mesoderm	3,21	6,63	1,33
BMP10	Mesoderm	111,32	264,25	21,54
CDH5	Mesoderm	25,86	3.381,32	15,19
CDX2	Mesoderm	6,60	13,44	5,34
COLEC10	Mesoderm	10,68	0,82	3,15
ESM1	Mesoderm	68,94	8,96	19,25
FCN3	Mesoderm	8,69	14,21	0,29
FOXF1	Mesoderm	25,80	70,37	3,14
HAND1	Mesoderm	49,83	144,92	34,07
HAND2	Mesoderm	183,24	242,43	18,45
HEY1	Mesoderm	7,99	107,20	9,13
HOPX	Mesoderm	125,69	131.646,05	34,24
IL6ST	Mesoderm	12,05	12,50	4,29
NKX2-5	Mesoderm	8,41	1,69	1,61
ODAM	Mesoderm	14,39	752,49	7,16
PDGFRA	Mesoderm	4,56	1,93	1,64
PLVAP	Mesoderm	18,24	5,26	1,05
RGS4	Mesoderm	69,10	71,92	7,93
SNAI2	Mesoderm	235,71	28,52	41,22
TBX3	Mesoderm	5,81	190,57	9,69
TM4SF1	Mesoderm	72,18	22,96	6,56
CXCL5	Self-renewal	0,23	0,07	0,87
DNMT3B	Self-renewal	0,00	0,04	0,01

HESX1	Self-renewal	0,02	0,02	0,02
IDO1	Self-renewal	0,00	0,03	0,00
LCK	Self-renewal	0,00	0,05	0,01
NANOG	Self-renewal	0,00	0,06	0,04
POU5F1	Self-renewal	0,02	0,13	0,08
SOX2	Self-renewal	0,18	0,02	0,18
TRIM22	Self-renewal	0,32	0,20	0,29

Experiment Filename		
**Target Name**	**Category**	**Keratinocyte**
CDH9	Ectoderm	0,50
COL2A1	Ectoderm	0,14
DMBX1	Ectoderm	0,42
DRD4	Ectoderm	0,27
EN1	Ectoderm	32,66
LMX1A	Ectoderm	0,76
MAP2	Ectoderm	1,49
MYO3B	Ectoderm	26,88
NOS2	Ectoderm	0,20
NR2F1/NR2F2	Ectoderm	0,25
NR2F2	Ectoderm	17,67
OLFM3	Ectoderm	5,33
PAPLN	Ectoderm	1,76
PAX3	Ectoderm	11,03
PAX6	Ectoderm	0,40
POU4F1	Ectoderm	4,19
PRKCA	Ectoderm	1,03
SDC2	Ectoderm	3,44
SOX1	Ectoderm	1,77
TRPM8	Ectoderm	4,45
WNT1	Ectoderm	11,42
ZBTB16	Ectoderm	0,69
AFP	Endoderm	0,26
CABP7	Endoderm	3,39
CDH20	Endoderm	1,37
CLDN1	Endoderm	3,31
CPLX2	Endoderm	0,56
ELAVL3	Endoderm	2,16
EOMES	Endoderm	1,53
FOXA1	Endoderm	0,45
FOXA2	Endoderm	0,75
FOXP2	Endoderm	8,91
GATA4	Endoderm	1,35
GATA6	Endoderm	0,70
HHEX	Endoderm	0,07
HMP19	Endoderm	2,54

HNF1B	Endoderm	3,36
HNF4A	Endoderm	0,21
KLF5	Endoderm	89,05
LEFTY1	Endoderm	1,13
LEFTY2	Endoderm	7,04
NODAL	Endoderm	0,19
PHOX2B	Endoderm	6,81
POU3F3	Endoderm	0,37
PRDM1	Endoderm	2,07
RXRG	Endoderm	14,37
SOX17	Endoderm	22,06
SST	Endoderm	14,66
FGF4	Mesendoderm	0,61
GDF3	Mesendoderm	4,36
NPPB	Mesendoderm	0,81
NR5A2	Mesendoderm	155,79
PTHLH	Mesendoderm	33,61
T	Mesendoderm	0,09
ABCA4	Mesoderm	28,43
ALOX15	Mesoderm	1,22
BMP10	Mesoderm	14,04
CDH5	Mesoderm	37,38
CDX2	Mesoderm	6,26
COLEC10	Mesoderm	22,04
ESM1	Mesoderm	3,90
FCN3	Mesoderm	1,10
FOXF1	Mesoderm	5,87
HAND1	Mesoderm	257,23
HAND2	Mesoderm	4,03
HEY1	Mesoderm	3,16
HOPX	Mesoderm	1.856,77
IL6ST	Mesoderm	8,06
NKX2-5	Mesoderm	27,79
ODAM	Mesoderm	518,18
PDGFRA	Mesoderm	1,56
PLVAP	Mesoderm	1,14
RGS4	Mesoderm	7,03
SNAI2	Mesoderm	5,20
TBX3	Mesoderm	21,16
TM4SF1	Mesoderm	8,56
CXCL5	Self-renewal	5,17
DNMT3B	Self-renewal	0,04
HESX1	Self-renewal	1,44
IDO1	Self-renewal	1,11
LCK	Self-renewal	3,26
NANOG	Self-renewal	2,90
POU5F1	Self-renewal	1,75
SOX2	Self-renewal	2,88

TRIM22	Self-renewal	5,29

Fold change legend

fc > 100	Upregulated
10 < fc <= 100	
2 < fc <= 10	
0.5 <= fc <= 2	Comparable
0.1 <= fc < 0.5	
0.01 <= fc < 0.1	
fc < 0.01	Downregulated
omitted	

FACS Transfection Efficiency

A

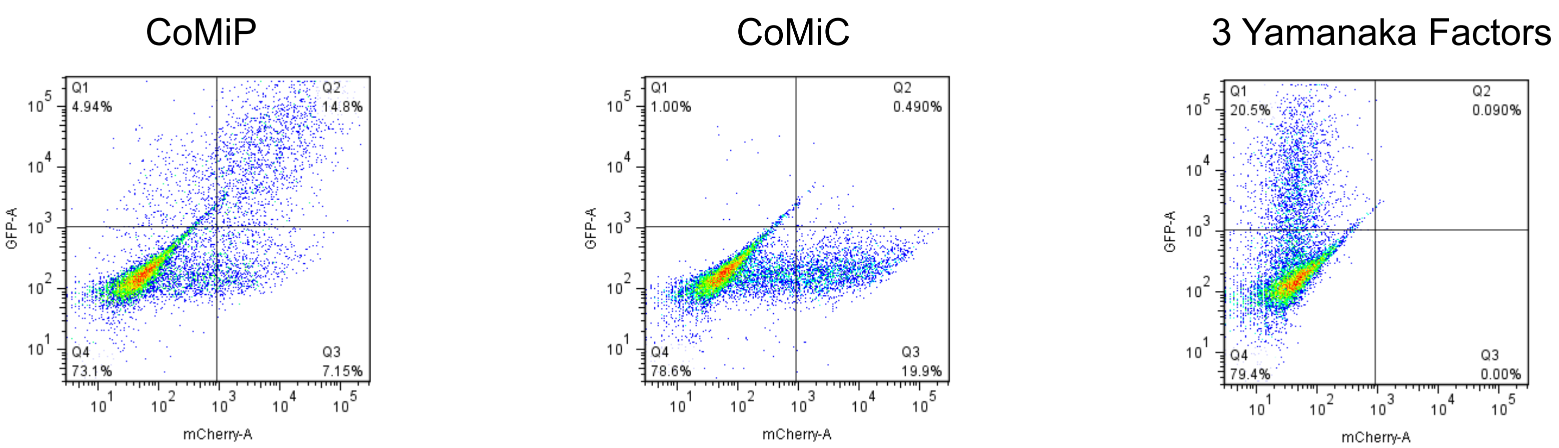

B

Reprogramming Plasmids	FACS positive cells			
	Sample 1	Sample 2	Average	STDEV
CoMiP	14.8	28.1	21.45	9.4
CoMiC	19.9	10.2	15.05	6.86
3 Yamanaka factors	20.5	12.2	16.35	5.87

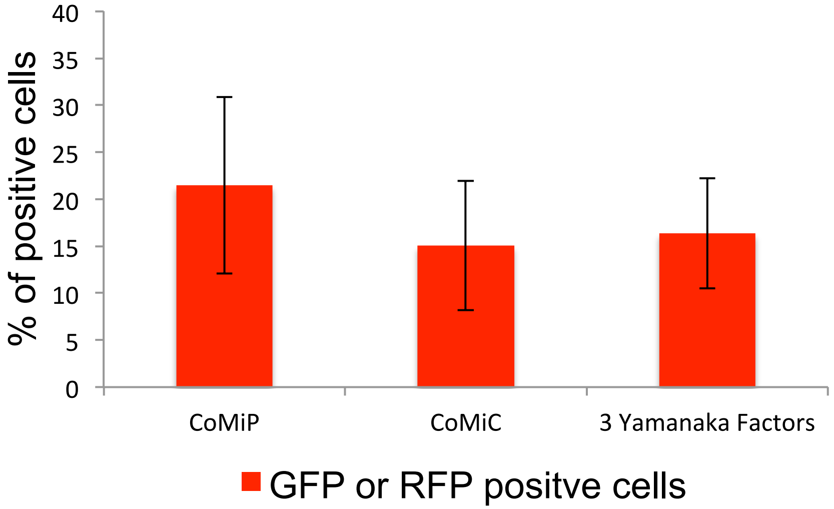

Sup Figure 1

FACS Viability – Propidium Iodide Staining

A

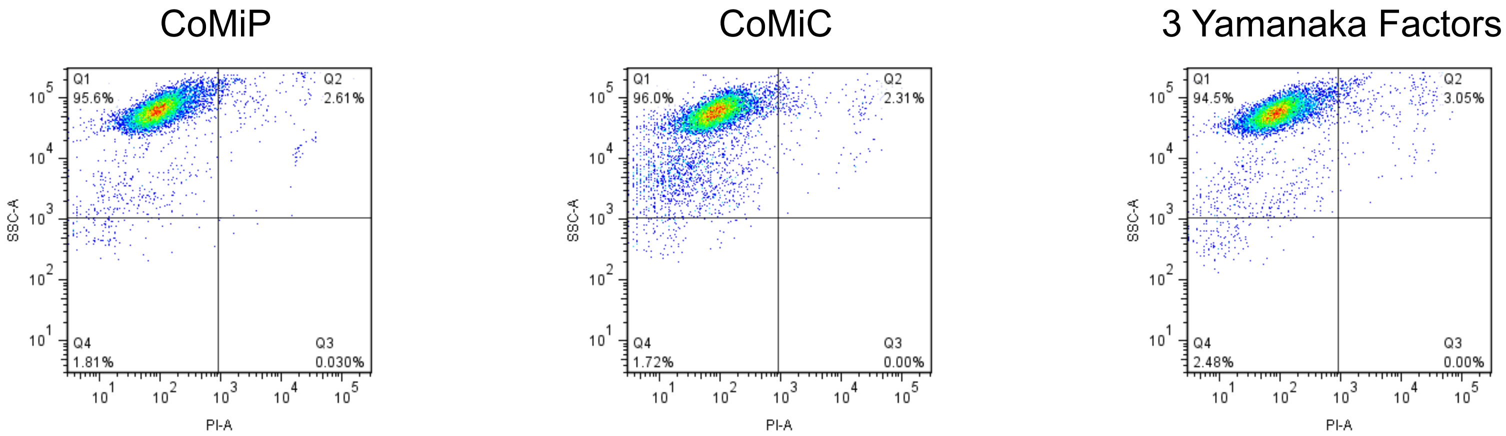

B

Reprogramming Plasmids	Cell number		FACS			
	Sample 1	Sample 2	Sample 1		Sample 2	
			PI negative	PI positive	PI negative	PI positive
CoMiP	245000	230000	96.9	3.1	97.8	2.2
CoMiC	167500	162500	97.7	2.3	97.4	2.6
3 Yamanaka factors	130000	140000	97.0	3.0	96.4	3.6

C

Reprogramming Plasmids	Relative number of cells alive after transfection (1100000 cell transfected)			
	Sample 1	Sample 2	Average	STDEV
CoMiP	21.58	20.45	21.02	0.80
CoMiC	11.55	12.39	11.97	0.60
3 Yamanaka factors	14.77	14.33	14.55	0.31

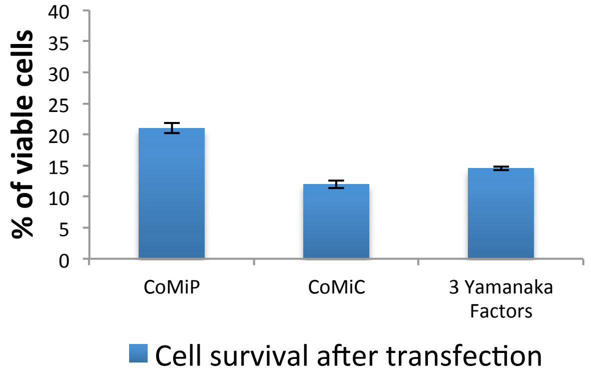

Sup Figure 2

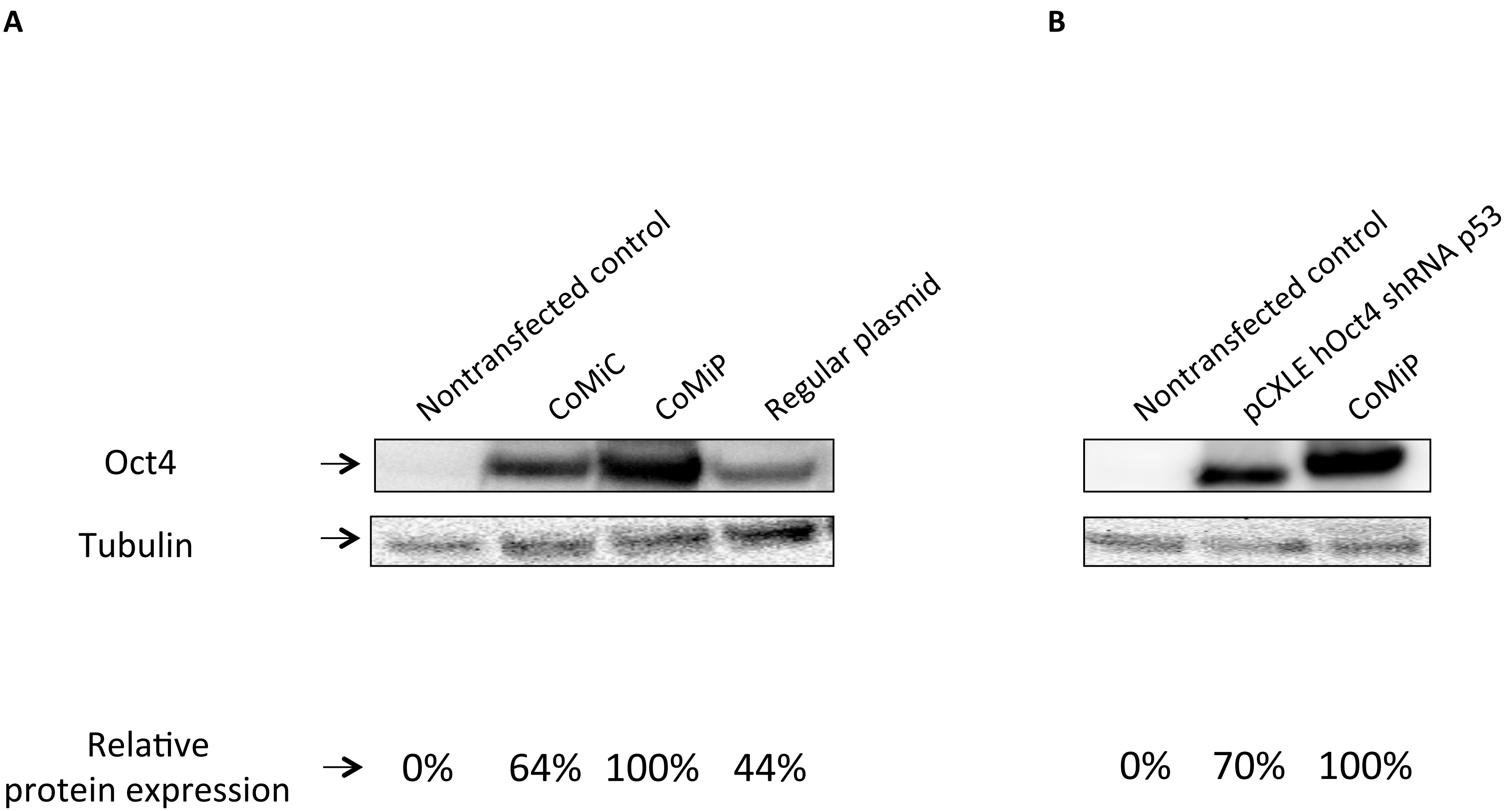
A
B
Nontransfected control
CoMiC
CoMiP
Regular plasmid
Oct4
Tubulin
Relative protein expression
0%
64%
100%
44%
Nontransfected control
pCXLE hOct4 shRNA p53
CoMiP
0%
70%
100%

Sup Figure 3

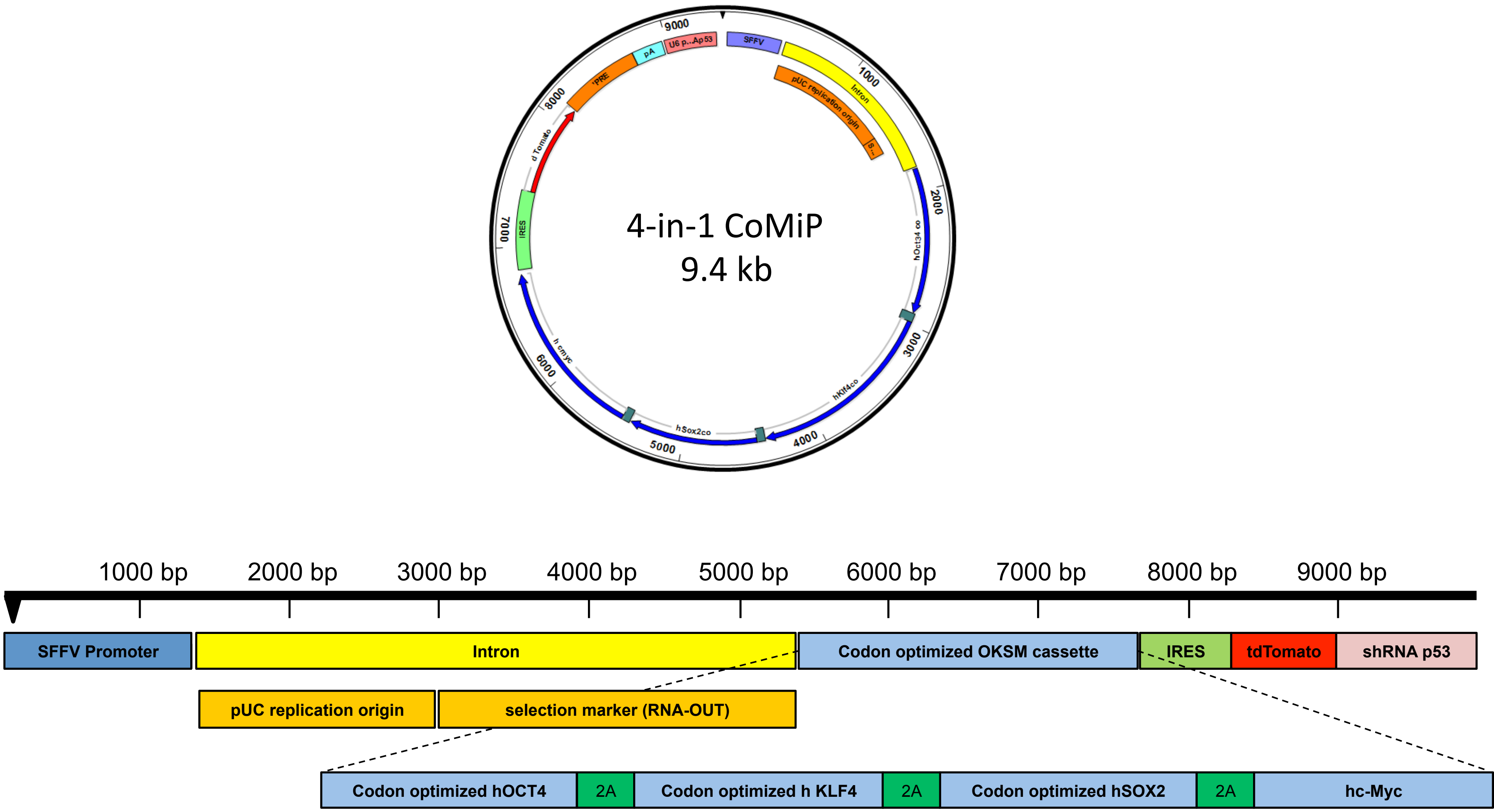
4-in-1 CoMiP
9.4 kb
SFFV
Intron
pUC replication origin
hOct34 co
hKlf4co
hSox2co
h cmyc
IRES
pA
U6 p...Ap53
1000
2000
3000
4000
5000
6000
7000
8000
9000
1000 bp
2000 bp
3000 bp
4000 bp
5000 bp
6000 bp
7000 bp
8000 bp
9000 bp
SFFV Promoter
Intron
Codon optimized OKSM cassette
IRES
tdTomato
shRNA p53
pUC replication origin
selection marker (RNA-OUT)
Codon optimized hOCT4
2A
Codon optimized h KLF4
2A
Codon optimized hSOX2
2A
hc-Myc

Sup Figure 4

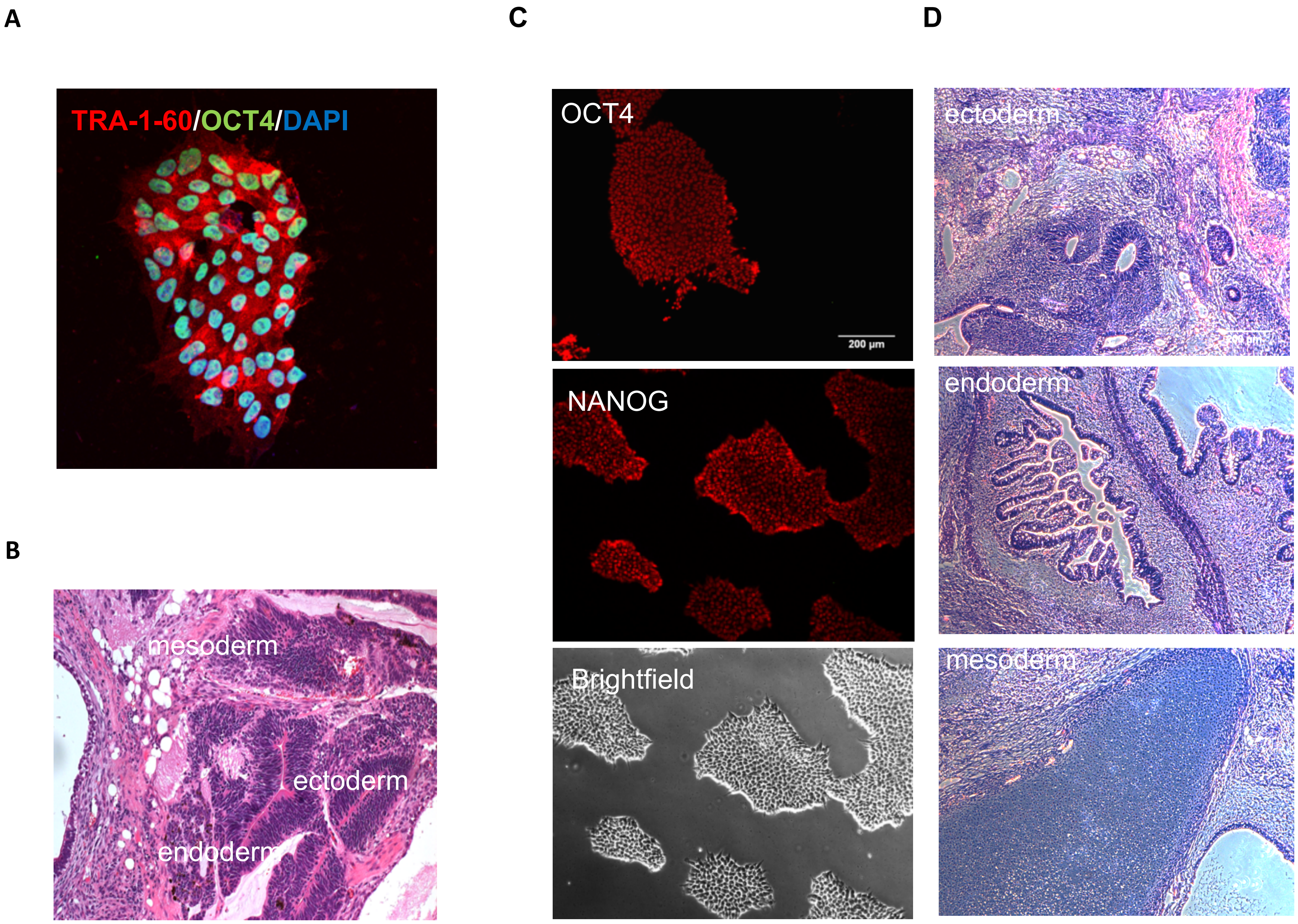
A
TRA-1-60/OCT4/DAPI
B
mesoderm
ectoderm
endoderm
C
OCT4
200 µm
NANOG
Brightfield
D
ectoderm
endoderm
mesoderm

Sup Figure 5

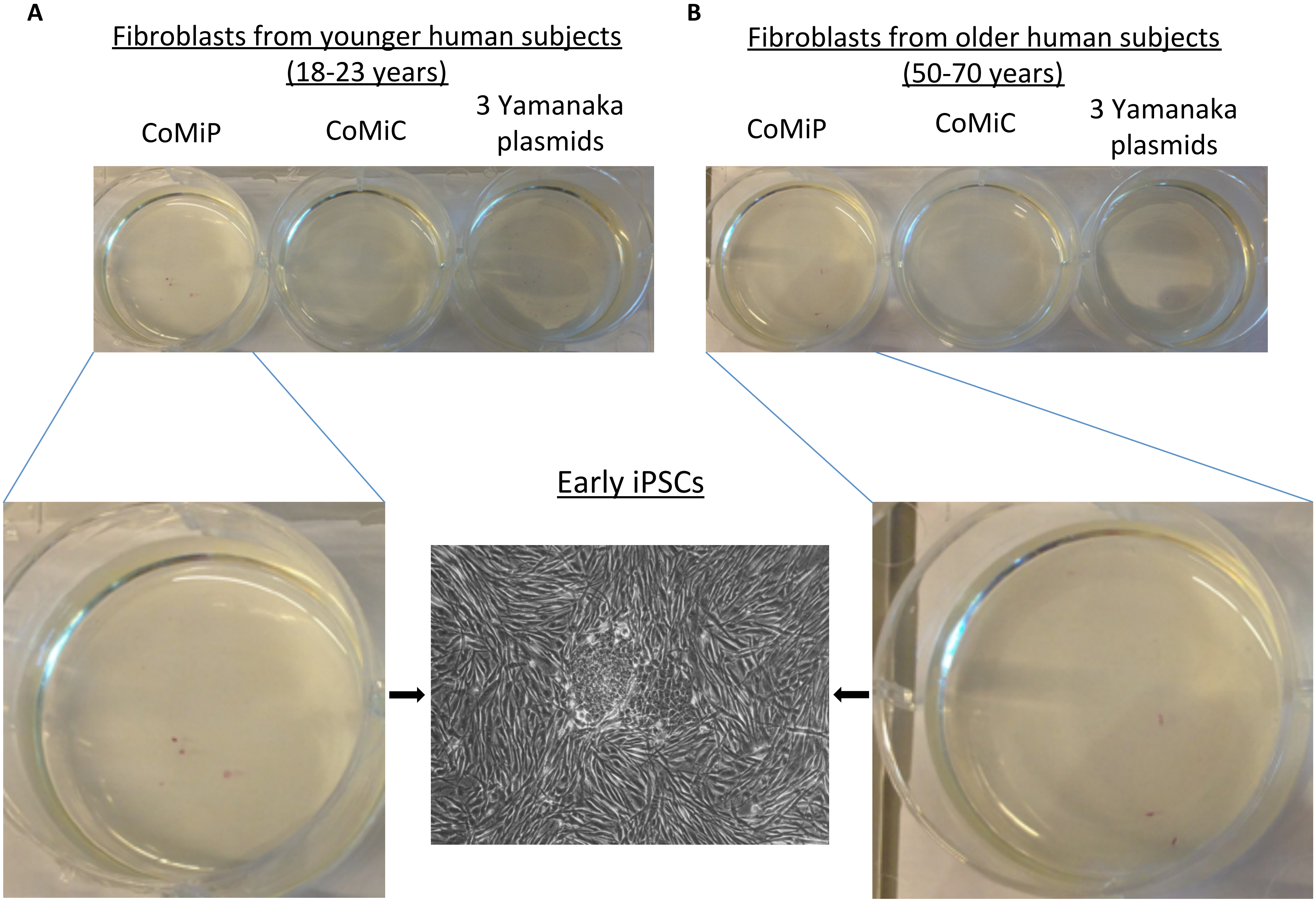
A
Fibroblasts from younger human subjects
(18-23 years)
CoMiP
CoMiC
3 Yamanaka plasmids
B
Fibroblasts from older human subjects
(50-70 years)
CoMiP
CoMiC
3 Yamanaka plasmids
Early iPSCs

Sup Figure 6

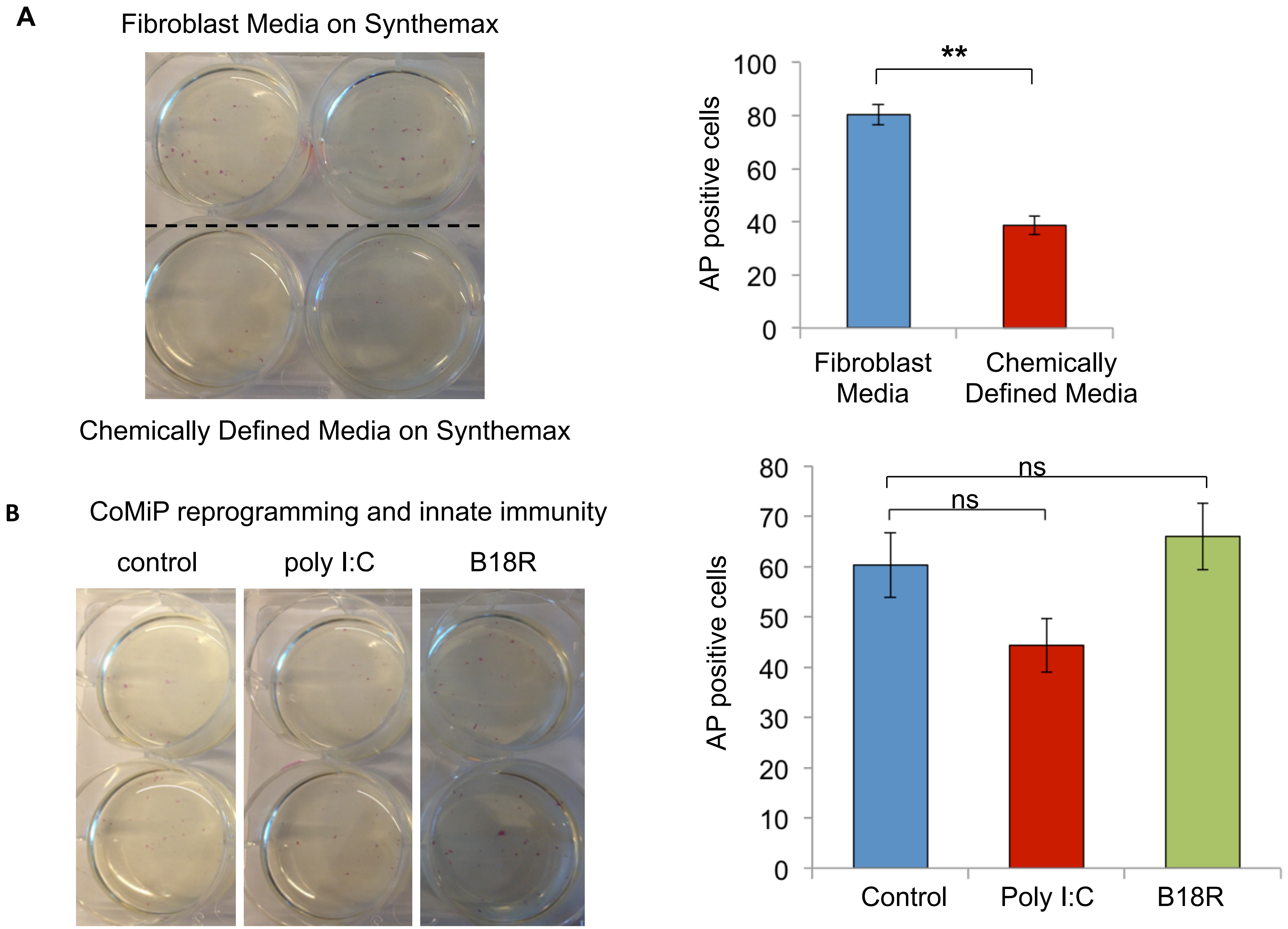
A
Fibroblast Media on Synthemax
Chemically Defined Media on Synthemax
**
AP positive cells
100
80
60
40
20
0
Fibroblast Media
Chemically Defined Media
B
CoMiP reprogramming and innate immunity
control
poly I:C
B18R
ns
ns
AP positive cells
80
70
60
50
40
30
20
10
0
Control
Poly I:C
B18R

Sup Figure 7

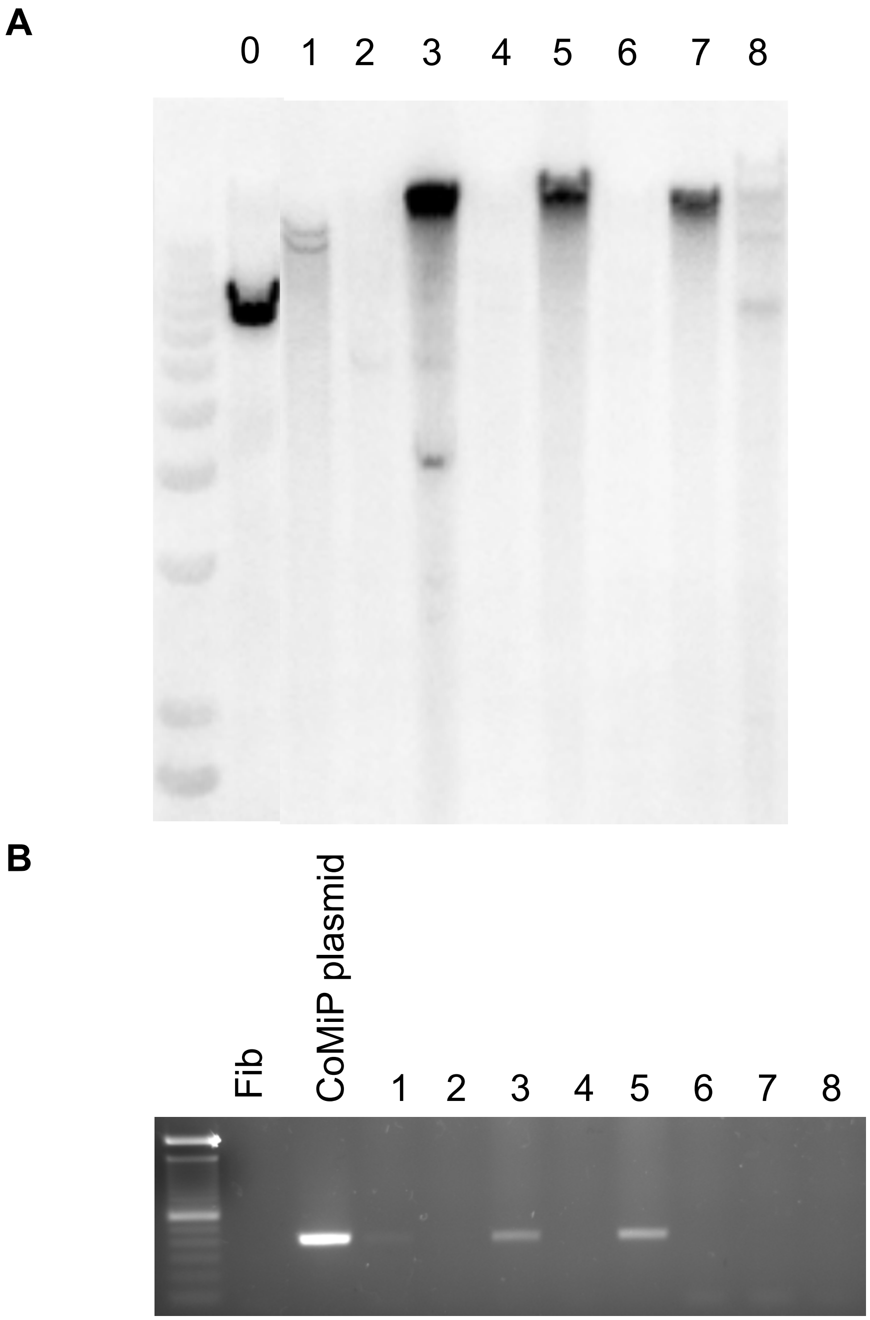

	Marker
0	Fibroblasts + CoMiP digested
1	hiPSC clone 1 (Lentivirus)
2	miPSC clone 1 (CoMiP, Electroporation)
3	miPSC clone 2 (CoMiP, Electroporation)
4	hiPSC clone 1 (CoMiP, Electroporation)
5	hiPSC clone 2 (CoMiP, Electroporation)
6	hiPSC clone 3 (CoMiP, Electroporation)
7	hiPSC clone 4 (CoMiP, Lipofection)
8	hiPSC clone 5 (CoMiP, Lipofection)

Sup Figure 8

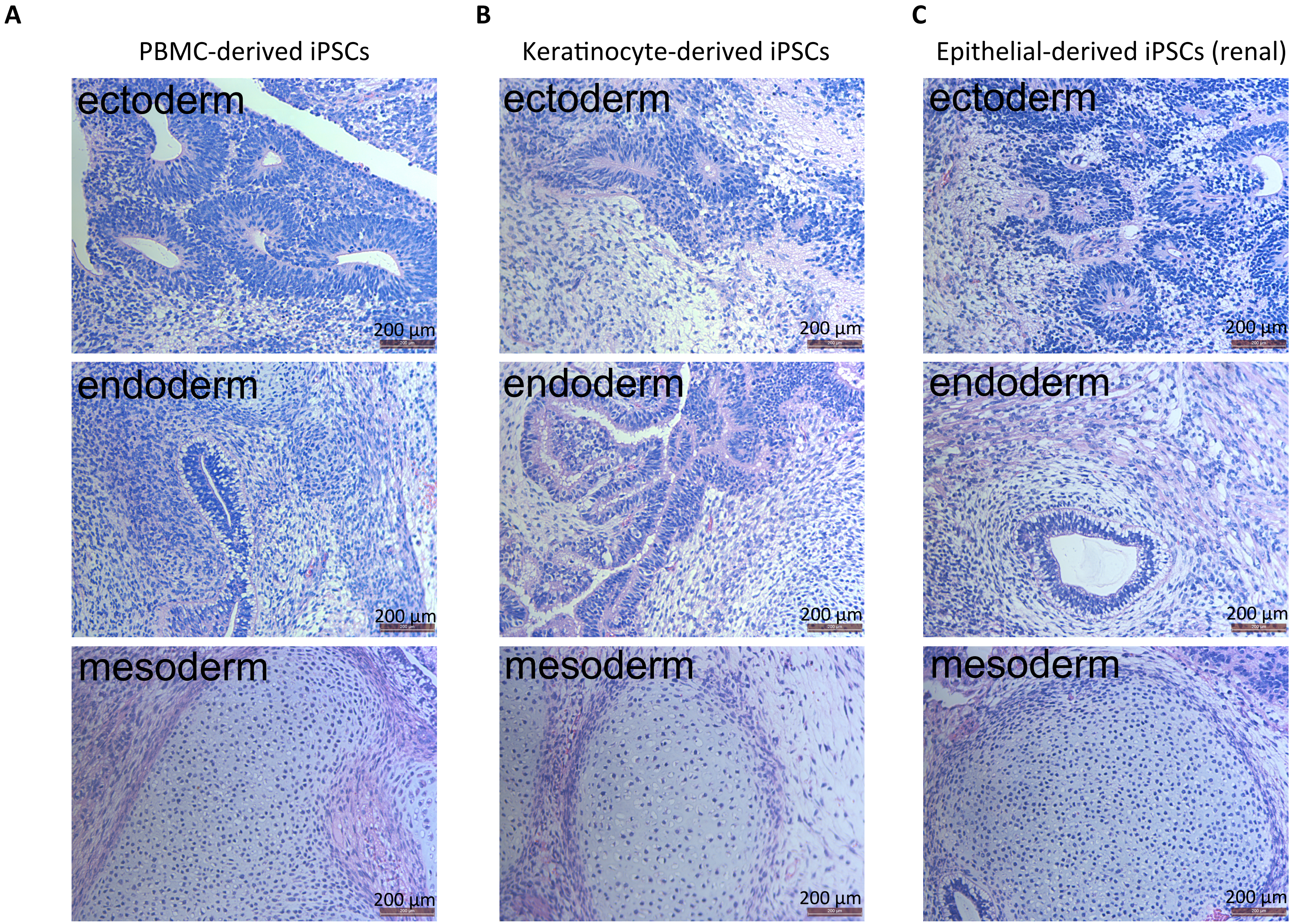
A
PBMC-derived iPSCs
B
Keratinocyte-derived iPSCs
C
Epithelial-derived iPSCs (renal)
ectoderm
endoderm
mesoderm
200 µm

Sup Figure 9

Scorecard Pluripotency Evaluation

A

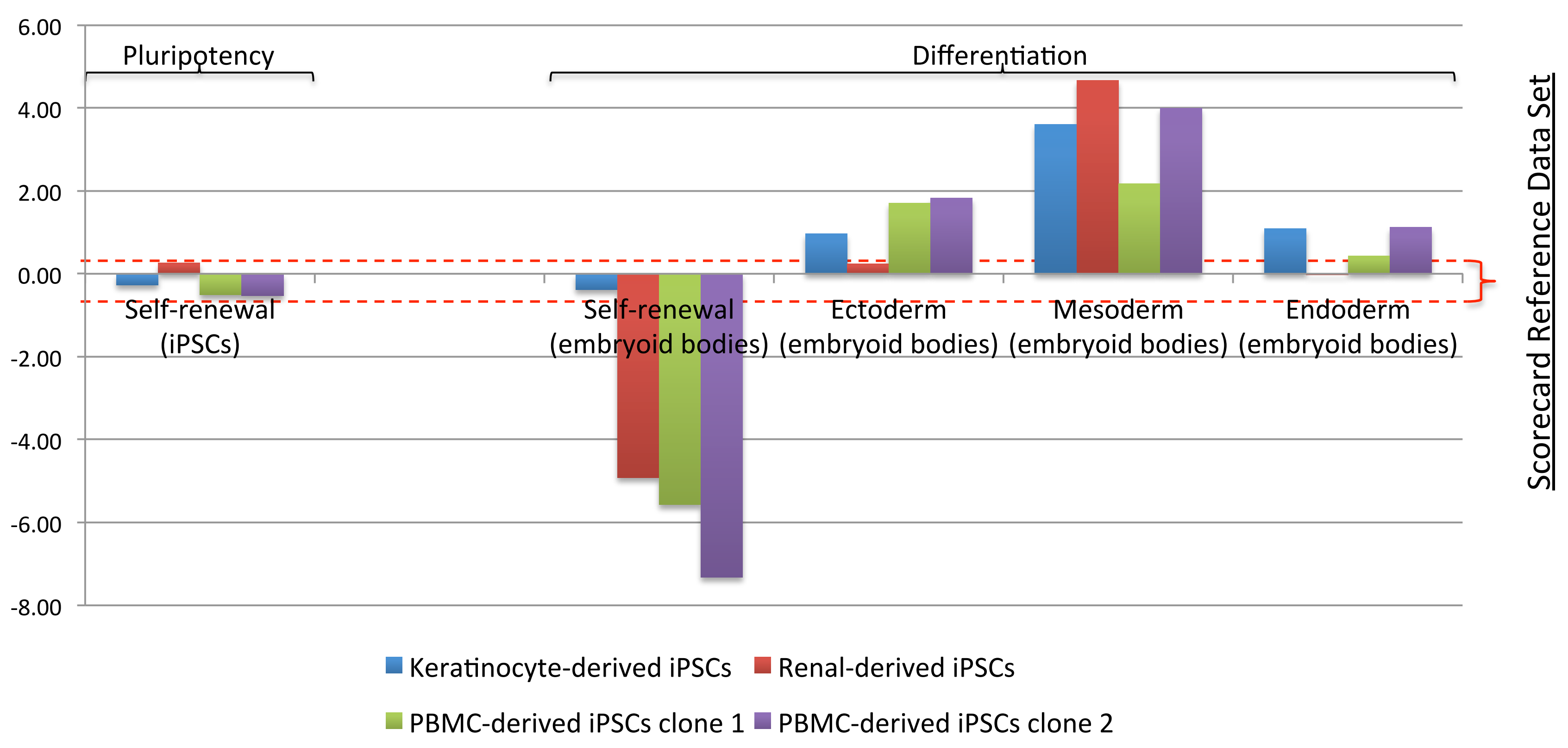

B

iPSC line	Ectoderm Germ layer	Mesoderm Germ layer	Endoderm Germ layer
Keratinocyte-derived iPSCs	0,97	3,61	1,09
Renal-derived iPSCs	0,24	4,67	-0,03
PBMC-derived iPSCs clone 1	1,71	2,17	0,43
PBMC-derived iPSCs clone 2	1,83	4,00	1,12

Differentiation propensity

High

Comparable

Low

Sup Figure 10

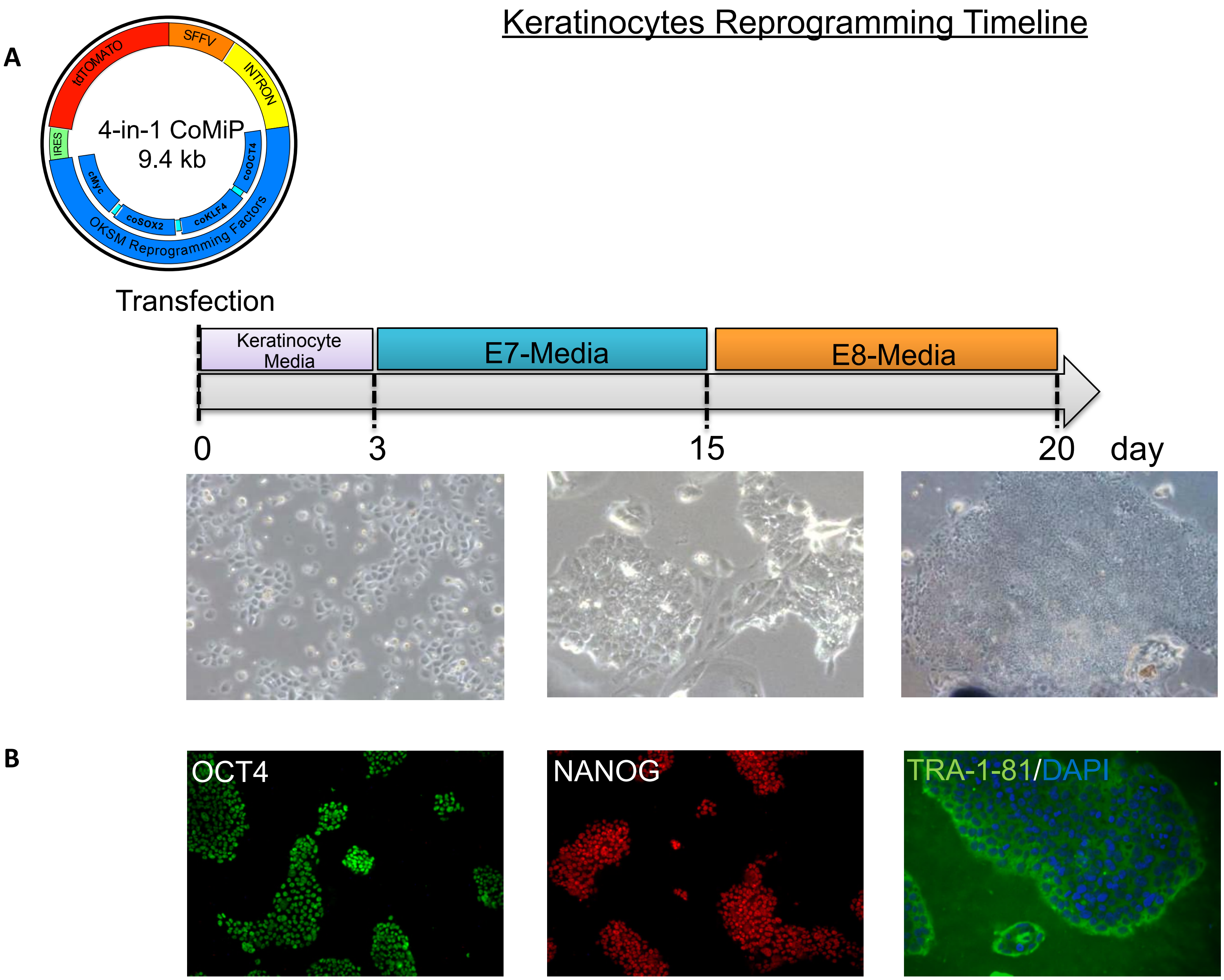

Sup Figure 11

Mouse iPSCs

A

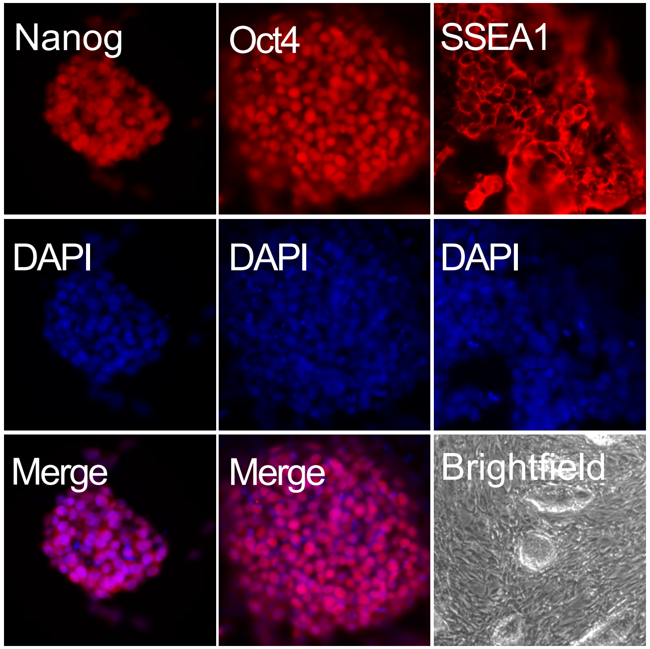

B

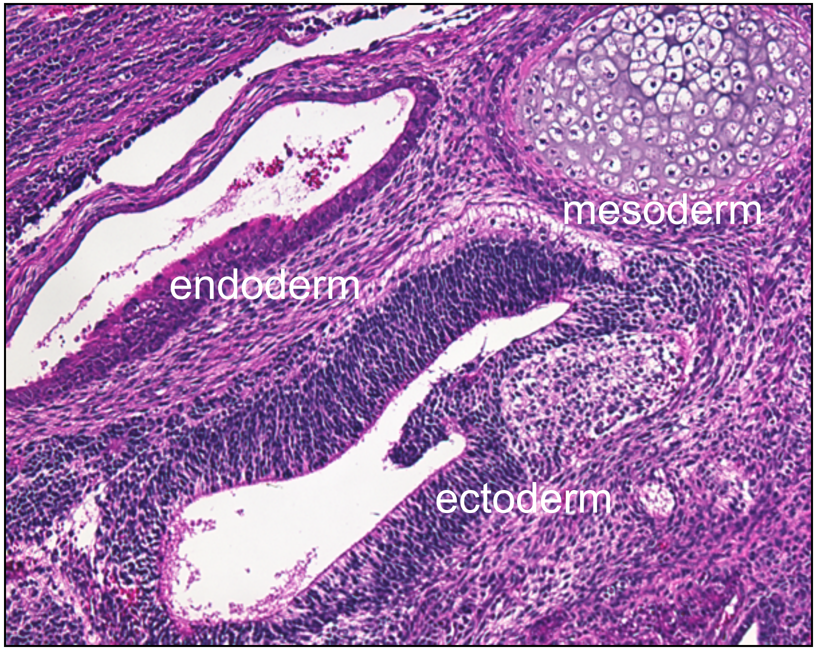

Sup Figure 12

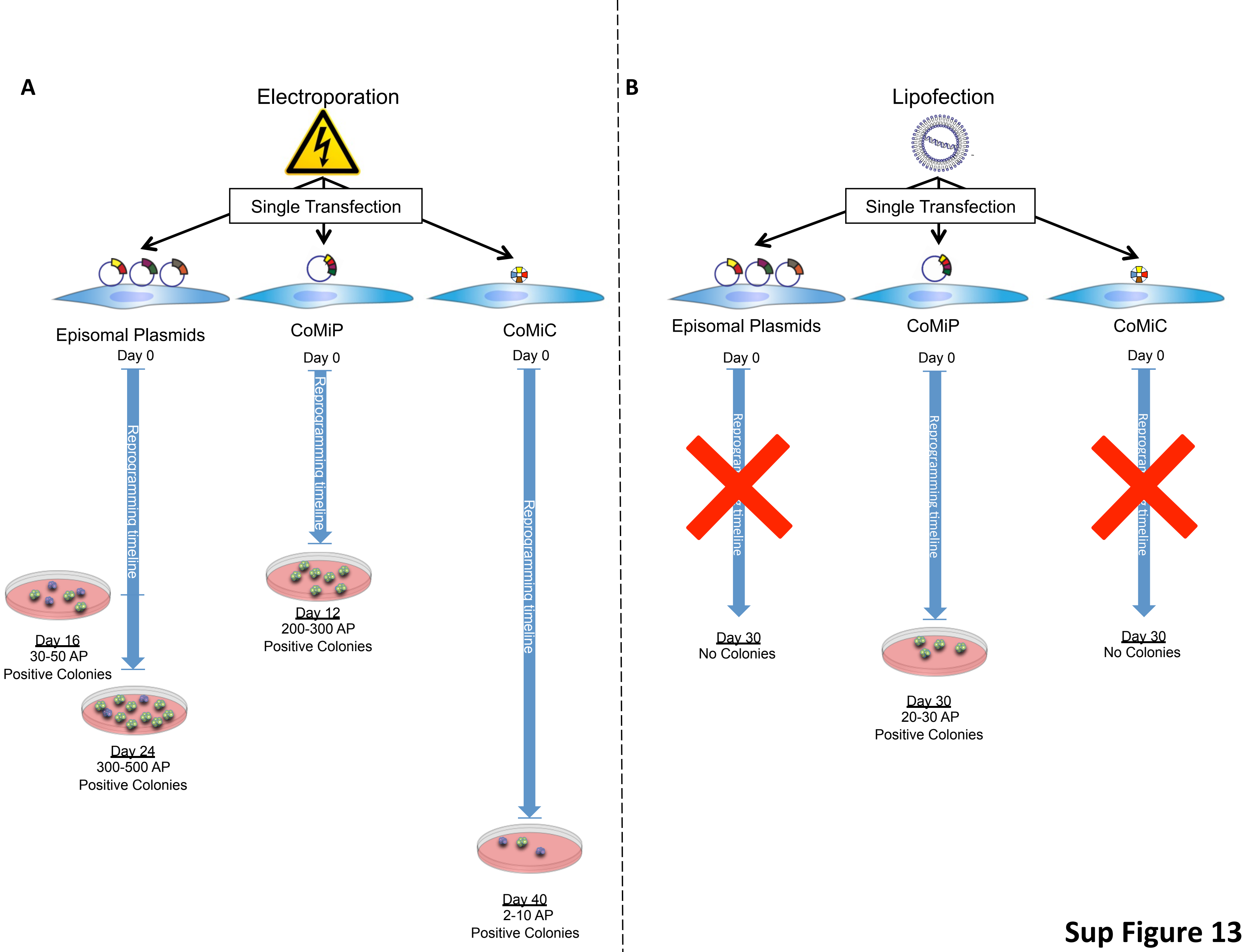

Sup Figure 13
